# Gasdermin D deficiency aggravates nephrocalcinosis-related chronic kidney disease with rendering macrophages vulnerable to necroptosis

**DOI:** 10.1038/s41419-025-07620-1

**Published:** 2025-04-13

**Authors:** Yoshihiro Kusunoki, Chenyu Li, Hao Long, Kanako Watanabe-Kusunoki, Meisi Kuang, Julian Aurelio Marschner, Andreas Linkermann, Stefanie Steiger, Hans-Joachim Anders

**Affiliations:** 1 Renal Division, Department of Medicine IV, Hospital of the Ludwig-Maximilians-University, Munich, Germany; 2 Department of Rheumatology, Endocrinology, and Nephrology, Faculty of Medicine and Graduate School of Medicine, Hokkaido University, Sapporo, Japan; 3 Department of Medicine, Renal Electrolyte and Hypertension Division, Perelman School of Medicine, University of Pennsylvania, Philadelphia, PA USA; 4 Department of Urology, The Affiliated Hospital of Southwest Medical University, Luzhou, China; 5 Department of Pharmacy, Ludwig-Maximilians-University, Munich, Germany; 6 Department of Medicine V, University Medical Centre Mannheim, University of Heidelberg, Mannheim, Germany; 7 Department of Internal Medicine 3, University Hospital Carl Gustav Carus at the Technische Universität Dresden, Dresden, Germany; 8 Division of Nephrology, Department of Medicine, Albert Einstein College of Medicine, Bronx, NY USA

**Keywords:** Necroptosis, Chronic kidney disease

## Abstract

Several forms of regulated necrosis contribute to the pathogenesis of crystal nephropathy, however, the role of pyroptosis, an inflammatory form of cell death involving the formation of gasdermin-D pores in internal and external cell membranes, in this condition remains unknown. Our transcriptional and histological analyses suggest that Gsdmd in tubulointerstitital cells may contribute to the pathogenesis of chronic oxalate nephropathy. However, genetic deletion of *Gsdmd* exacerbated oxalate nephropathy in mice in association with enhanced CaOx crystal deposition and accelerated tubular epithelial cell injury. Pharmacological inhibition of necroptosis reversed this effect. Indeed, *Gsdmd*^*−/−*^ bone marrow-derived macrophages were more prone to undergo necroptosis when stimulated with CaOx crystals compared to their wildtype counterparts. We conclude that gasdermin D suppresses the necroptosis pathway, which determines the outcome of oxalate nephropathy-related nephrocalcinosis.

## Introduction

Crystal formation inside kidney tubules can cause various types of kidney injury depending on their localization and size. In both urolithiasis and intrarenal nephrocalcinosis, calcium oxalate (CaOx) crystals are most prevalent [1]. Large kidney stones sometimes lodge in the ureter, leading to hydronephrosis and subsequent kidney injury. In contrast, nephrocalcinosis itself does not cause hydronephrosis but can lead to crystal-induced nephropathy and kidney failure.

CaOx crystal-induced nephropathy involves crystal formation from filtered minerals inside the tubules due to supersaturation of calcium and oxalate, combined with insufficient crystallization inhibitors in the urine. These formed crystals lead to subacute plug formation in the tubules, followed by an inflammatory response due to pro-inflammatory cytokines released from inflammatory cell or as a result of cell death, ultimately leading to loss of kidney function [2].

The cellular uptake of CaOx crystals into tubular epithelial cells is known to induce necroptosis, a form of regulated cell death that contributes to kidney dysfunction [2]. In addition to necroptosis, pyroptosis is another regulated form of cell death, involving the formation of gasdermin D (GSDMD) pores into membranes of the cell as the key executer of this pathway [3–5]. Pyroptosis has been implicated in several kidney diseases [6–10]. Whether inhibiting this cell death pathway could lead to favorable outcomes remains a topic of debate. In CaOx crystal-induced acute nephropathy, intrarenal CaOx crystals can activate the NLRP3 inflammasome, a key component in pyroptosis, in kidney-resident dendritic cells [11], while also directly triggering tubular cell necroptosis, both contributing to kidney injury [12]. In the context of CaOx crystal-induced chronic nephropathy, Knauf et al. demonstrated that CaOx crystal deposition triggers NLRP3-mediated inflammation, which contributes to kidney dysfunction [13]. Our group reported additional inflammasome-independent functions of NLRP3 that promote kidney fibrosis in a mouse model of nephrocalcinosis-related CKD [14]. Thus, the involvement of NLRP3 in CaOx-induced crystal nephropathy is established. However, the role of pyroptosis in this context remains controversial.

GSDMD, along with most other members of gasdermin family, is responsible for the final step of pyroptosis, facilitating the release of IL-1β from inside the cell into the surrounding environment [6], and is considered essential for pyroptosis. As described earlier, while the involvement of NLRP3 in CaOx crystal-induced chronic nephropathy has already been demonstrated [13, 14], the effect of inhibiting GSDMD in this context remains unclear. We speculated that *Gsdmd* deletion would elicit a renoprotective effect similar to that of *NLRP3* deletion in CaOx crystal-induced chronic nephropathy but our experimental studies revealed unexpected results.

## Methods and Materials

### Animal studies

*Gsdmd*^*−/−*^ mice were kindly provided by Prof. Dr. Andreas Linkermann (Mannheim, Germany). All mice were housed in groups of five in filter-top cages and had access to food and water *ad libitum*. Cages, nestlets, food, and water were sterilized by autoclaving before use. Eight-week-old *Gsdmd*^*−/−*^ mice and WT mice received oxalate-rich diet for 21 days. An oxalate-rich diet was prepared by adding 50 μmol/g sodium oxalate to a calcium-free standard diet (Ssniff, Soest, Germany) as previously described [15]. Mice were sacrificed by cervical dislocation at day 21 after starting the oxalate-rich diet. Plasma and urine samples were collected and GFR was measured at different time points before sacrifice and harvesting the kidneys. One part of each kidney was fixed in formalin and subsequently embedded in paraffin for histological analysis, another part was kept in RNA later solution at -20 °C, and the rest part was kept at -80 °C for immunoblotting.

### Transcutaneous measurement of glomerular filtration rate (GFR)

GFR measurements were performed in conscious mice on day -1, 7, 14, and 20. Briefly, mice were anesthetized with isoflurane to mount a miniaturized imager device built from two light-emitting diodes, a photodiode and a battery (MediBeacon, Mannheim, Germany) onto the shaved neck of the animals [16]. The background signal of the skin was recorded for 5 min. Then, mice received an intravenous injection of 150 mg/kg FITC-sinistrin (MediBeacon). Each mouse was conscious and kept in a single cage, and the signal was recorded for 90 min. Data were analyzed using MPD Lab software (MediBeacon). The GFR [μl/min] was calculated from the decrease of fluorescence intensity over time using a two-compartment model, the animals body weight and an empirical conversion factor [16].

### Immunoblotting

Total protein from kidney tissues or cells was extracted in RIPA buffer (89900, Thermo Fisher Scientific, Waltham, MA, USA), which was supplemented with protease and phosphatase inhibitors (A32961, Thermo Fisher Scientific). Insoluble components were removed by centrifugation at 12 000 × g for 10 min, and protein concentration was determined by Bradford assay (Bio-Rad, Hercules, CA, USA). Protein was denatured using Laemmli buffer and 2-mercaptoethanol, and boiled for 5 min at 95 °C. Samples were separated by 10% SDS-PAGE and were transferred to polyvinylidene fluoride membranes. The following primary antibodies were used: mouse anti-mouse caspase-1 (AG-20B-0042-C100, Adipogen, San Diego, CA, USA), mouse anti-human/mouse IL-1β (#12242, Cell Signaling Technology, Danveers, MA, USA), rabbit anti-mouse GSDMD (ab219800, Abcam, Cambridge, United Kingdom), rabbit anti-DFNA5/GSDMD antibody (ab215191, Abcam), rabbit anti-Receptor interacting protein kinase (RIPK) (#3493, Cell Signaling Technology), rabbit anti-Receptor interacting protein kinase-3 (RIPK3) (ab62344, Abcam), rat anti-Mixed lineage kinase domain like pseudokinase (MLKL) (MABC604, Sigma-Aldrich), and rabbit anti-β actin (#4967, Cell Signaling Technology). The following secondary antibodies were used: anti-rabbit IgG-HRP (#7074, Cell Signaling Technology) and anti-mouse IgG-HRP (#7076, Cell Signaling Technology). The blot was visualized using an enhanced chemiluminescence substrate on a ChemiDoc Imaging System (Bio-Rad).

### Immunofluorescence staining for macrophages

Bone marrow-derived macrophages (BMDMs) were fixed with 4% paraformaldehyde for 10 min and permeabilized with 0.2% TritonX-100 for 5 min. Subsequently, the cells were blocked and incubated with primary antibodies, including rabbit anti-pMLKL (37333, Cell Signaling Technology) and rat anti-F4/80 (NB600-404, NOVUS Biologicals, Littleton, CO, USA). The macrophages were then treated with secondary antibodies, including Alexa Fluor 488 goat anti-rabbit IgG (Jackson ImmunoResearch, West Grove, PA) and Cy3 donkey anti-rat IgG (Jackson ImmunoResearch). 4’,6-diamidino-2-phenylindole (DAPI) solution (0.1 μg/ml, NBP2-31156, NOVUS Biologicals) was added for nuclear staining. Fluorescent imaging was performed using a Nikon Eclipse Ti2 microscope (NIKON, Tokyo, Japan).

### CaOx crystal formation in vitro

The in vitro formation of CaOx crystals was performed, as previously described [17]. Briefly, 10 mM sodium oxalate (Na_2_C_2_O_4_) and 10 mM calcium chloride (CaCl_2_) solutions were prepared along with a crystallization buffer (90 mM Tris-HCL, 10 mM NaCl). A 5 mM Na_2_C_2_O_4_ (pH 7.3) was pre-incubated with or without 10 μl of urine for 1 h, followed by a 3 h incubation with 1 mM CaCl_2_. The size and area occupied by the formed crystals were assessed using a Nikon Eclipse Ti2 microscope (NIKON, Tokyo, Japan). The number of crystals was determined using FACSCalibur (Becton Dickinson, New Jersey, USA), and analyzed with FlowJo version 10 software (Tree Star, Ashland, OR, USA).

### Statistical analysis

Statistical analysis was performed using GraphPad Prism 7 software (Graphpad, La Jolla, CA, USA). In vivo data are presented as the mean ± standard deviation (SD) using Student’s t-test to determine statistical significances between two groups. For comparing three or more groups, we applied one-way analysis of variance (ANOVA) with Turkey’s post-hoc test. When two parameters with multiple groups were employed, we used two-way ANOVA with Bonferroni’s multiple comparisons test. In vitro data are presented as the mean ± standard error of the mean (SEM) using one-way ANOVA with Dunnett’s multiple comparisons test to compare three or more groups. Two or more parameters with multiple groups we compared using two-way ANOVA with Dunnett’s multiple comparisons test. Statistical significance was determined by *P* values of less than 0.05, which were indicated as *p < 0.05, **p < 0.01, and ***p < 0.001.

For further details on additional experimental and analytical methods please consult the online supplement.

## Results

### Transcriptional and histological analyses show Gsdmd expression in tubulointerstitial cells in chronic oxalate nephropathy

**Fig. 1:**
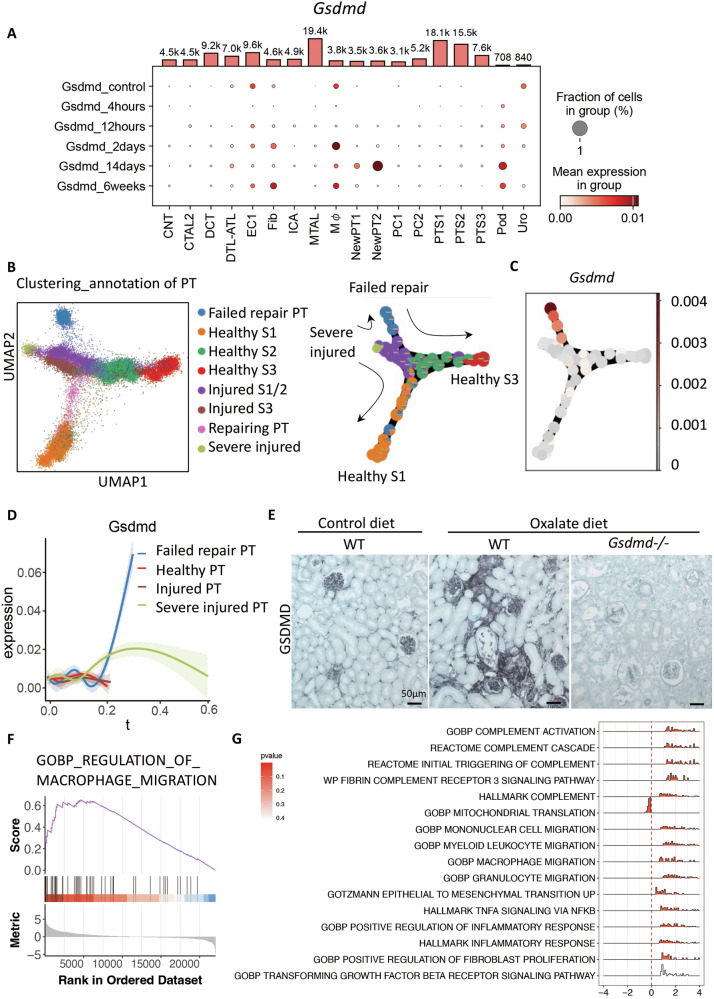
Gsdmd is expressed in macrophages, glomerular cells, and presumably in injured proximal tubular cells that fail to undergo repair in the kidney, and gene sets related to macrophage migration are upregulated in a chronic oxalate nephropathy mice model. **A** Dot plot representing the expression of Gsdmd in each kind of cells in the kidney. The dot color indicates the average gene expression level in each cluster, while the dot size represents the percentage of cells in each cluster. The bar plots on the top of the box are the numbers of cells in each group. **B** Pseudotime trajectory of proximal tubular cells in uIRI mouse model, colored by cluster identity. **C** Expression levels of Gsdmd in each cluster of proximal tubular cells seven days after uIRI in trajectory analysis. **D** Supervised pseudotime analysis of *Gsdmd* gene expression levels in each cluster of proximal tubular cells after uIRI. The x-axis represents pseudotime, and the y-axis shows z-scored gene expression values. **E** Representative images of Gsdmd staining in the kidneys of WT mice fed a control diet, and WT and *Gsdmd*^*−/−*^ mice fed an oxalate-rich diet. **F** The enrichment plots from the GSEA analysis show gene enrichment profiles comparing WT mice fed an oxalate-rich diet versus a control diet. The plots highlight the enrichment of transcriptional signatures related to macrophage migration. **G** Density ridge plots showing the distribution of gene expression for core-enriched genes in enriched gene sets, with gradient colors indicating adjusted p-values using the Benjamini-Hochberg method. CNT: connecting tubule, CTAL2: thick ascending limb of loop of Henle in cortex, DCT: distal convoluted tubule, ATL: thin ascending limb of loop of Henle, EC: endothelial cells, Fib: fibroblasts, ICA: type A intercalated cells of collecting duct, MTAL: thick ascending limb of loop of Henle in medulla, Mφ: macrophages, PT: proximal tubule, PC: principle cells, PTS1: S1 segment of proximal tubule, PTS2: S2 segment of proximal tubule, PTS3: S3 segment of proximal tubule, Pod: Podocytes, Uro: Urothelium.

Gsdmd is known to be expressed in macrophages, and has a pivotal role in pyroptosis [18]. To investigate the gene expression of Gsdmd in injured kidney, we analyzed a previously published single-cell RNA sequencing dataset [19], which was based on cells sorted from the kidneys of C57BL/6 J mice before and after bilateral kidney ischemia-reperfusion injury. Gsdmd was expressed in kidney macrophages, and also in podocytes and endothelial cells under physiological conditions (Fig. 1A). Furthermore, a part of the proximal tubular cells−undergoing compensatory adaptation (“failed repair”) expressed Gsdmd during the chronic phase after ischemia-reperfusion injury (Fig. 1B–D). Consistently, immunostaining for Gsdmd in Wild type (WT) mice treated with an oxalate-rich diet showed positivity in interstitial cells, glomerular cells, and larger clusters of tubular epithelial cells, presumably corresponding to these aforementioned tubular cells undergoing adaption, which was not observed in healthy controls (Fig. 1E and Supplementary figure S1). The specificity of the staining of Gsdmd was proven by negative staining results in *Gsdmd*^*−/−*^ mice (Fig. 1E).

Furthermore, to investigate potential therapeutic targets in chronic oxalate nephropathy, we fed WT mice an oxalate-rich, calcium-depleted diet to induce progressive oxalate nephropathy [15] (Supplementary Fig. S2A), and performed RNA sequencing on their kidney lysates. Cluster analysis clearly separated mice fed a control diet from those fed an oxalate-rich diet (Supplementary Fig. S2B), and Gsdmd and other pyroptosis-related genes such as Casp1, IL-1β, and NLRP3 were detected as differentially expressed genes (Supplementary table S1). Furthermore, gene set enrichment analysis clarified significant changes in gene sets related to macrophage migration, chemokine pathways, in the chronic oxalate nephropathy model (Fig. 1F, G, Supplementary figure S2D-H). Gsdmd expression in macrophages and tubular cells in the kidney may contribute to the pathogenesis of chronic oxalate nephropathy.

### Loss of gasdermin D exacerbates kidney injury in CaOx crystal-induced chronic nephropathy

**Fig. 2:**
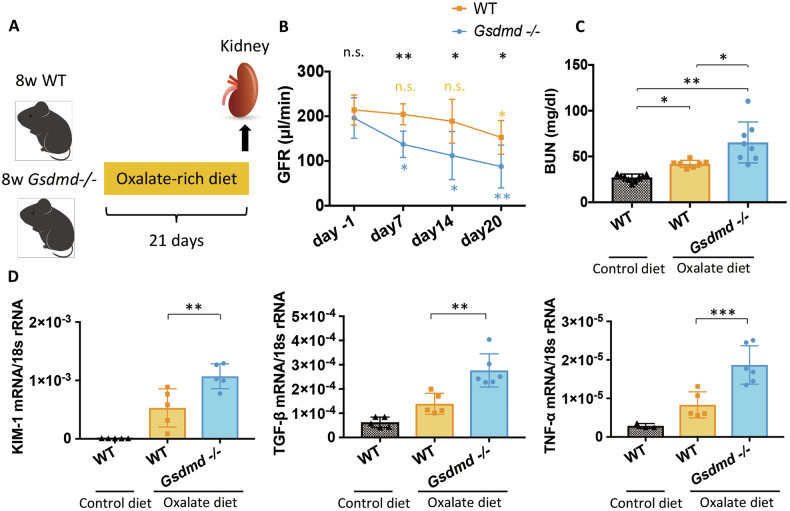
Gasdermin D knockout (*Gsdmd*^−/−^) mice subjected to an oxalate-rich diet manifest a worsened renal outcome in comparison to WT mice. **A** Study design illustration. Eight-week-old WT (*n* = 15) and *Gsdmd*^*−/−*^ mice (*n* = 13) received an oxalate rich diet for 21 days. The mice were sacrificed on day 21 for subsequent analyses. **B** Glomerular filtration rate (GFR) at various time points in WT and *Gsdmd*^*−/−*^ mice. **C** Blood Urea Nitrogen (BUN) levels on day 21 in WT mice fed a control diet and in WT and *Gsdmd*^*−/−*^ mice fed an oxalate-rich diet. **D** mRNA expression of KIM-1, TGFβ1, and TNFα in kidney RNA isolates. Data are presented as mean ± SD. *P* values were calculated using two-way ANOVA with Bonferroni’s multiple comparisons test (**B**), one-way ANOVA with Turkey’s post-hoc test (**C**), or Mann-Whitney U test (**D**). n.s., not significant; *P < 0.05, **P < 0.01, ***P < 0.001.

**Fig. 3:**
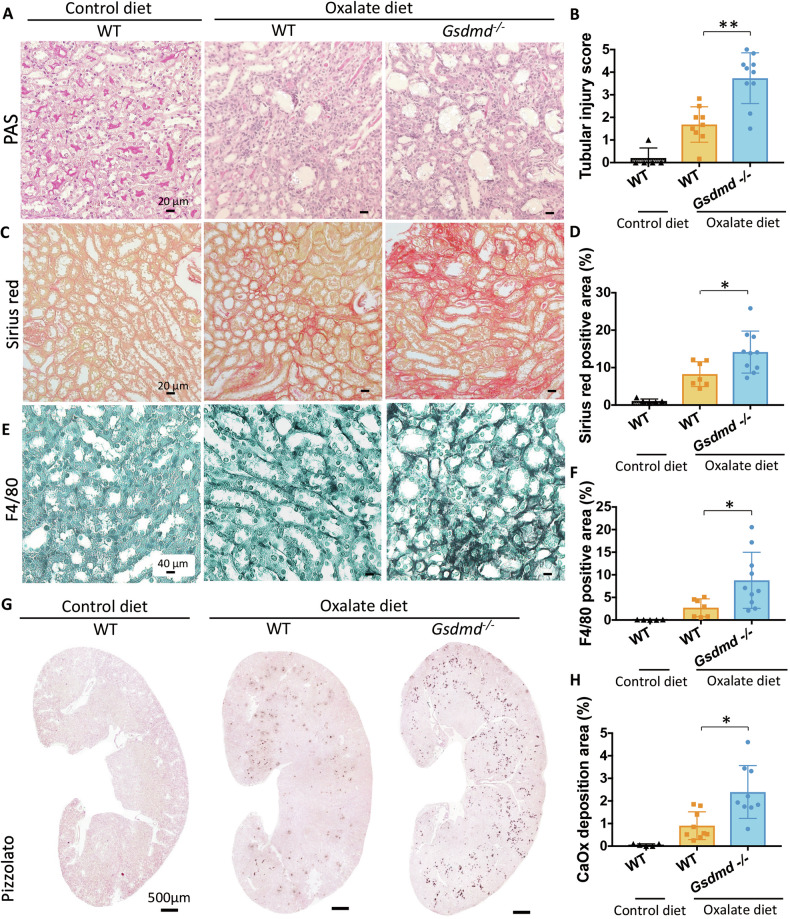
Renal histological analysis shows that genetic *Gsdmd* deletion exacerbates kidney injury in CaOx crystal-induced chronic oxalate nephropathy mouse model. **A** Periodic acid-Schiff (PAS) staining illustrating tubular injury. **B** Quantification of tubular injury. **C** Sirius red staining illustrating fibrosis in the kidney. **D** Quantification of Sirius red-positive areas. **E** Immunostaining showing F4/80 macrophages in the kidney. **F** Quantification of macrophage infiltration. **G** Pizzolato staining illustrating calcium oxalate (CaOx) crystal deposition in the kidney. **H** Quantification of Pizzolato-positive areas. Data are presented as mean ± SD. *P* values were calculated using Mann-Whitney U test. n.s., not significant; *P < 0.05, **P < 0.01.

To explore the possibility of a functional role of GSDMD in nephrocalcinosis-related kidney injury, we fed WT and *Gsdmd*^−/−^ mice an oxalate-rich, calcium-depleted diet to induce progressive oxalate nephropathy (Fig. 2A). By day 20, both *Gsdmd*^−/−^ and WT mice displayed significant oxaluria and hypocalciuria (Supplementary Fig, S3A and S3B). Neither *Gsdmd*^−/−^ nor WT mice developed albuminuria (Supplementary Fig. S3C), indicating that the disease primarily affected the tubulointerstitial compartment of the kidney, consistent with the chronic oxalate nephropathy model. Interestingly, starting from day seven, *Gsdmd*^−/−^ mice showed a faster decline in GFR compared to WT mice (Fig. 2B). This was accompanied by higher blood urea nitrogen (BUN) levels at day 20 (Fig. 2C). The more rapid decline in kidney excretory function in *Gsdmd*^−/−^ mice was associated with elevated kidney mRNA expression levels of kidney injury molecule-1 (KIM-1), transforming growth factor-beta 1 (TGF-β1), and tumor necrosis factor alfa (TNF-α) (Fig. 2D). Consistently, *Gsdmd*^−/−^ mice exhibited more severe tubular injury (Fig. 3A, B) and interstitial fibrosis (Fig. 3C, D), as well as increased interstitial macrophage infiltration (Fig. 3E, F) and nephrocalcinosis (Fig. 3G, H).

We also analyzed CaOx crystal-binding molecules CD44 and annexin II because we previously reported that induction of these molecules is a key element to link CaOx microcrystal formation with nephrocalcinosis and kidney injury [20]. In *Gsdmd*^−/−^ mice subjected to an oxalate-rich diet, the mRNA expression levels of CD44 and annexin II in the kidney (Supplementary Fig. S4A and S4B), coupled with the areas positively stained for these substances, were notably increased (Supplementary Fig. S4C-F).

These findings suggest that Gsdmd deficiency, either directly or indirectly, amplifies nephrocalcinosis-related tissue injury and remodeling, leading to a more rapid decline in kidney function. Additionally, the upregulation of the crystal binding molecules such as CD44 and annexin II may partially contribute to the exacerbation of kidney injury in *Gsdmd*^−/−^ mice within this model.

### Gasdermin D does not directly affect the formation of CaOx crystals in the urine

**Fig. 4:**
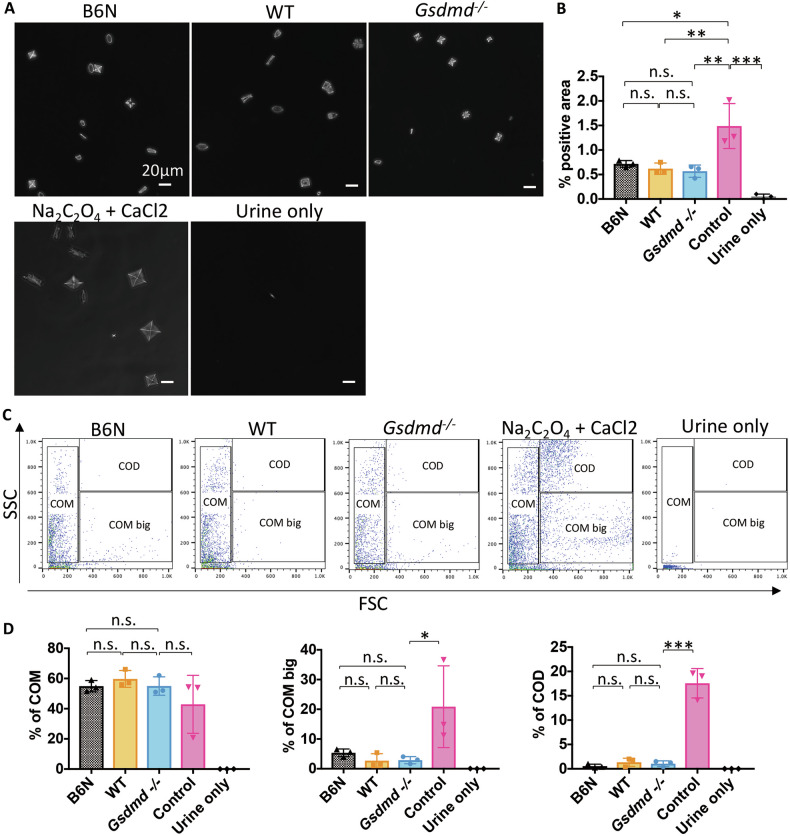
Genetic deletion of *Gsdmd* does not directly impact the formation of CaOx crystals under physiological conditions. **A** Representative phase-contrast microscope images of CaOx crystals observed 3 h after adding crystallization buffer, with or without urine from B6N, *Gsdmd* WT, and *Gsdmd* ^−/−^ mice, to supersaturated calcium and oxalate solutions. **B** Quantification of CaOx crystal-positive areas observed under a phase contrast microscopy. **C** Flow cytometry (forward scatter vs. sideward scatter) for assessing calcium oxalate monohydrate (COM) and calcium oxalate dihydrate (COD) crystal formation. Quantification of each type of crystals formed after 3 h of incubation with supersaturated calcium and oxalate solution buffer, with or without urine. Data are presented as mean ± SE from at least three independent experiments. *P* values were calculated using one-way ANOVA with Turkey’s post-hoc test (**B**, **D**). n.s., not significant; *P < 0.05, **P < 0.01, ***P < 0.001.

Gsdmd is secreted into the urine [21] hence it may directly affect CaOx crystal formation. In order to distinguish direct from indirect effects of GSDMD on nephrocalcinosis, we investigated whether *Gsdmd* deficiency affects the formation of CaOx crystals in urine by supersaturating calcium and oxalate in urine samples from each mouse genotype. CaOx crystal formation was assessed using phase-contrast microscopy and flow cytometry. Both methods revealed that urine from B6N, *Gsdmd* WT, and *Gsdmd*^*−/−*^ mice similarly reduced the formation of CaOx crystals, particularly large CaOx monohydrate crystals (COM big) and CaOx dihydrate crystals (COD), however, there were no significant differences between the urine samples from these three genotypes (Fig. 4A–D). This suggests that lack of GSDMD does not directly affect CaOx crystallization.

### Oxalate nephropathy in *Gsdmd*^−/−^ mice displays more necroptosis rather than less pyroptosis

**Fig. 5:**
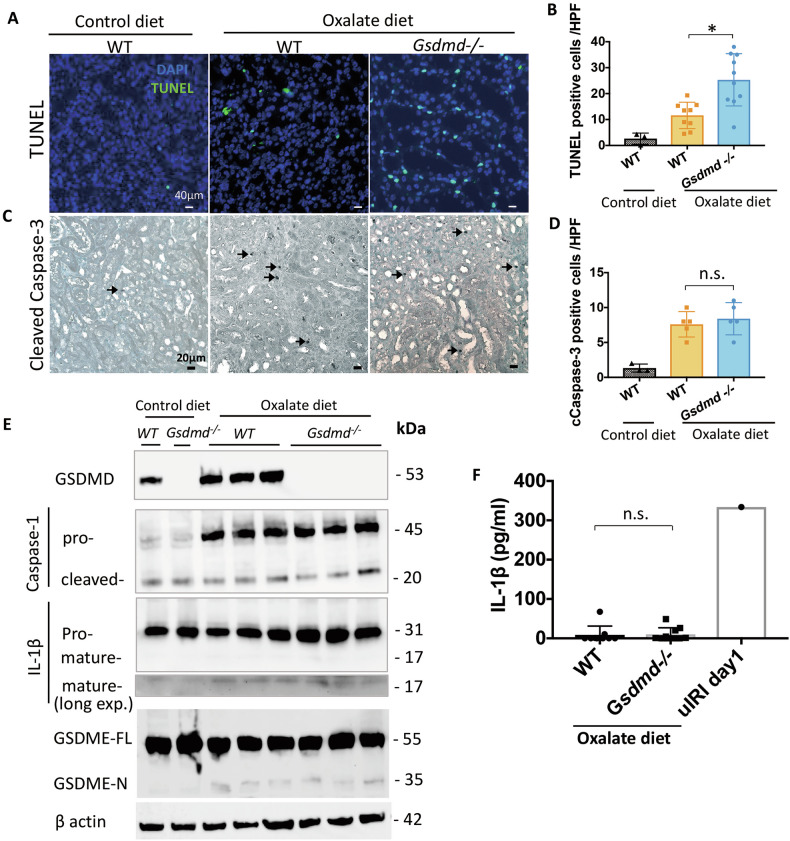
Immunobot analysis and serum IL-1β level suggest limited involvement of pyroptosis, while histological analysis shows no contribution of apoptosis to kidney injury in CaOx crystal-induced chronic nephropathy. **A** Representative TUNEL staining images from kidneys of WT mice fed a control diet, and of WT and *Gsdmd*^*−/−*^ mice fed an oxalate-rich diet. **B** Quantification of TUNEL positive cells. **C** Representative cleaved caspase-3 staining images from kidneys of WT mice fed a control diet, and of WT and *Gsdmd*^*−/−*^ mice fed an oxalate-rich diet. **D** Quantification of cleaved caspase-3 positive cells. **E** Immunoblot analysis of Gsdmd, caspase-1 (pro and cleaved p20), IL-1β (pro and mature p17), Gsdme (pro and cleaved p35) in the kidneys of WT and *Gsdmd*^*−/−*^ mice treated with an oxalate-rich diet. β-actin was used as a loading control. **F** IL-1β levels in serum from WT and *Gsdmd*^*−/−*^ mice fed an oxalate-rich diet. uIRI: unilateral ischemia-reperfusion injury. Data are presented as mean ± SD. *P* values were calculated using one-way ANOVA with Turkey’s post-hoc test (**B**, **D**, **F**). n.s., not significant; *P < 0.05.

Nephrocalcinosis triggers various forms of regulated kidney cell necrosis [12, 22]. TUNEL staining revealed a higher number of positive cells in the kidney of *Gsdmd*^−/−^ mice with oxalate nephropathy compared to WT mice, indicating increased kidney cell death (Fig. 5A, B) and cleaved caspase-3 staining showed apoptosis being unaffected by Gsdmd deficiency (Fig. 5C, D).

GSDMD is a known component of the pyroptosis pathway. However, immunoblot analysis of kidney lysates showed no discernible differences in the expression of other components in this pathway, such as cleaved caspase-1 (p20) and mature IL-1β (p17), between *Gsdmd*^−/−^ mice on an oxalate-rich diet and their WT counterparts on the same diet (Fig. 5E). Furthermore, the concentration of IL-1β in the serum exhibited no significant difference between mice with an oxalate-rich diet, irrespective of their *Gsdmd* genotype (Fig. 5F). This may be because either pyroptosis rarely occurs in this model or, if it does, a process called “delayed IL-1β release” is taking place. According to this concept, macrophages release IL-1β in a Gsdmd-independent manner several hours after stimulation with the pyroptosis inducer nigericin, although not immediately [23, 24]. Similarly, in our BMDMs, IL-1β release levels in *Gsdmd*^*−/−*^ BMDMs increased to nearly equivalent levels several hours after nigericin stimulation (data not shown). In our chronic oxalate nephropathy model, this delayed release mechanism may explain why both WT and *Gsdmd*^*−/−*^ mice ultimately achieve equivalent IL-1β release. Regarding the lack of difference in pyroptosis-related markers in the kidney between WT and *Gsdmd*^*−/−*^ mice in the Western blot analysis, this may be because Gsdmd is primarily responsible for IL-1β release during the pyroptosis process. Therefore, its presence or absence may not influence the expression levels of intracellular IL-1β and caspases in this model.

**Fig. 6:**
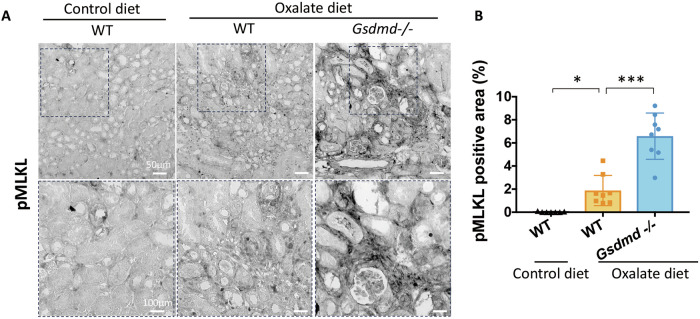
Histological and immunoblot analyses enhanced induction of necroptosis in *Gsdmd*^−/−^ mice within CaOx crystal-induced chronic nephropathy. **A** Representative images of pMLKL staining in kidneys from WT mice fed a control diet, and of WT and *Gsdmd*^*−/−*^ mice fed an oxalate-rich diet. The bottom panels show higher magnification views of the regions outlined by dotted rectangles in the top panels. **B** Quantification of pMLKL-positive areas. Data are presented as mean ± SE. *P* values are calculated using one-way ANOVA with Turkey’s post-hoc test. ***P < 0.001.

On the other hand, Gasdermin E (GSDME) can compensate for GSDMD-deficiency and permit IL-1β release following inflammasome activation [24]. Therefore, we examined the protein expression of Gsdme. However, there was no significant difference in Gsdme expression (Fig. 5E), indicating the absence of a compensatory increase in Gsdme expression. These findings collectively indicate that there are no apparent differences in pyroptosis between *Gsdmd*^*−/−*^ mice and WT mice in this model, possibly due to delayed IL-1 β release, and the differences in the kidney phenotype between WT and *Gsdmd*^*−/−*^ mice might be owing to pyroptosis-independent function of Gsdmd. In contrast, the kidneys of *Gsdmd*^−/−^ mice showed greater positivity for pMlkl, implicating an increased presence of necroptotic cells in *Gsdmd*^*−*/−^ mice subjected to an oxalate-rich diet compared to their WT counterparts (Fig. 6A, B). Immunoblot analysis revealed augmented expression of necroptosis markers, including Ripk1, Ripk3 and Mlkl, in the kidney of *Gsdmd*^*−*/−^ mice fed an oxalate-rich diet (Supplementary Fig. S5). These findings may suggest that GSDMD regulates necroptosis during oxalate nephropathy.

### Necroptosis inhibition improves oxalate nephropathy in *Gsdmd*^−/−^ mice

**Fig. 7:**
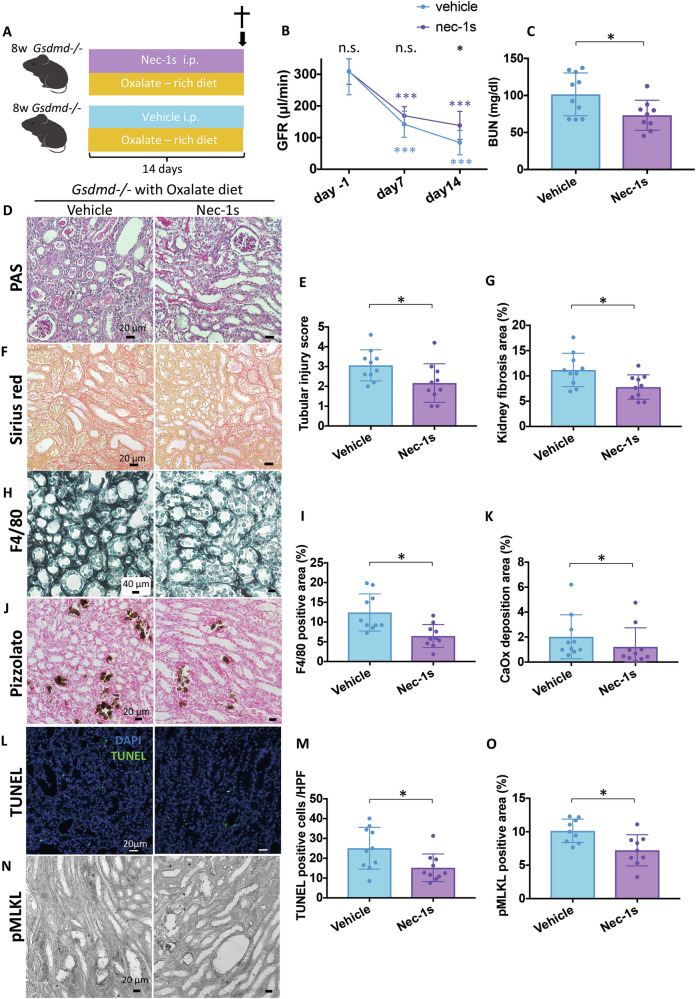
Inhibiting necroptosis ameliorates renal outcomes in *Gsdmd*^−/−^ mice within CaOx crystal-induced chronic nephropathy. **A** Study design illustration. *Gsdmd*^*−/−*^ mice were fed an oxalate-rich diet with or without Nec-1s for 14 days. Mice were sacrificed on day 14 for subsequent analysis. (n = 10 mice/group) (**B**) GFR at different time points in *Gsdmd*^*−/−*^ mice. **C** BUN levels on day 14 in *Gsdmd*^*−/−*^ mice. **D** PAS staining illustrating tubular injury in the kidneys. **E** Quantification of tubular injury. **F** Sirius red staining illustrating fibrosis in the kidney. **G** Quantification of Sirius red-positive areas. **H** Immunostaining illustrating F4/80 macrophages in the kidney. **I** Quantification of macrophage infiltration. **J** Pizzolato staining illustrating CaOx crystal deposition in the kidney. **K** Quantification of Pizzolato-positive areas. **L** Representative images of TUNEL staining in kidneys. **M** Quantification of TUNEL-positive cells. N Representative images of pMLKL staining on kidneys. **N** Quantification of pMLKL-positive area. Data are presented as mean ± SE. P values are calculated using two-way ANOVA with Bonferroni’s multiple comparisons test (**B**) or Mann-Whitney U test (**C**, **E**, **G**, **I**, **K**, **M**, **O**). n.s., not significant; *P < 0.05.

To explore the role of necroptosis in this context, we fed *Gsdmd*^*−/−*^ mice an oxalate-rich diet and treated them with a specific RIPK1 inhibitor, Nec-1s. All mice were sacrificed on day 14 (Fig. 7A). Notably, Nec-1s treatment had beneficial effects on kidney function, as evidenced by a diminished decline in GFR and lower BUN levels at the end of the study (Fig. 7B, C). Histological analysis further revealed that Nec-1s treatment mitigated tubular injury, interstitial fibrosis, macrophage infiltration, and nephrocalcinosis (Fig. 7D-K), along with TUNEL-positive cell death, including markers of necroptosis (Fig. 7L-O). These findings suggest that necroptosis contributes to the aggravated oxalate nephropathy phenotype observed in *Gsdmd*^*−/−*^ mice.

### *Gsdmd*^*−/−*^ macrophages are more susceptible to necroptosis upon stimulation with CaOx crystals

**Fig. 8:**
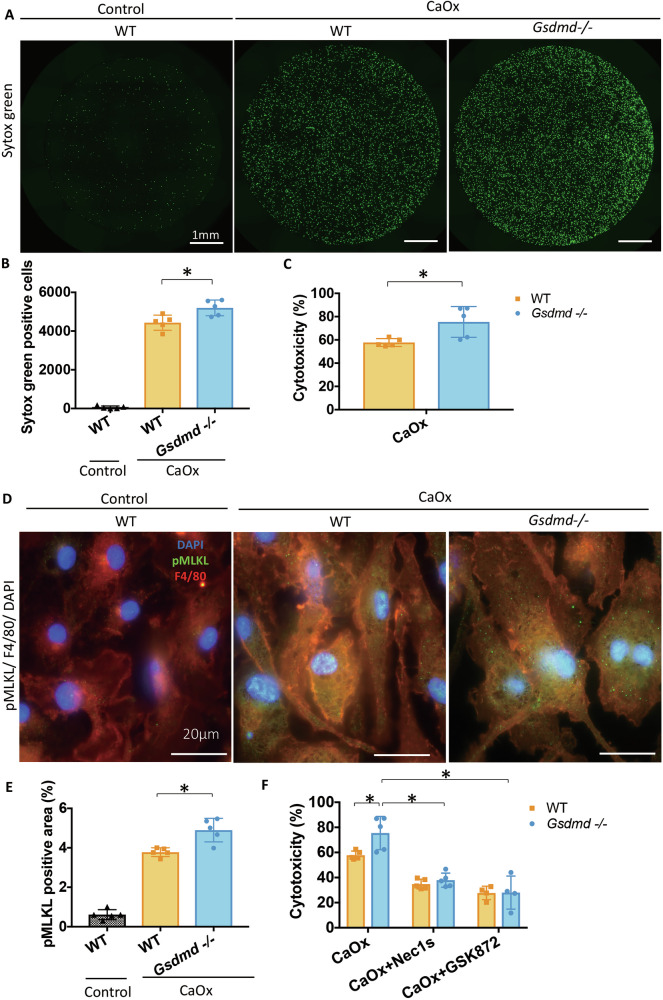
*Gsdmd*^*−/−*^ macrophages are vulnerable to CaOx crystal-induced damage, leading to an elevated occurrence of necroptosis. **A** Representative images of BMDMs stained with SYTOX Green (SG) 4 h after stimulation with CaOx crystals. **B** Quantification of SG-positive cells at 4 h after stimulation with CaOx crystals. **C** BMDMs were stimulated with 1 000 μg/ml CaOx crystals for 18 h, and cell-free supernatants were collected for LDH assay. **D** Representative immunofluorescent images of BMDMs 18 h after stimulation with 1 000 μg/ml CaOx crystals. Green: pMLKL, Red: F4/80, Blue: 4’,6-diamidino-2-2phenylindole (DAPI). **E** Quantification of pMLKL positive areas in BMDMs treated with CaOx for 18 h. **F** After pretreatment with or without Nec-1s or GSK872 for 1 h, BMDMs were stimulated with 1 000 μg/ml CaOx crystals for 18 h. Cell-free supernatants were collected for LDH assay. Data are presented as mean ± SE from at least 3 independent experiments. P values are calculated using one-way ANOVA with Turkey’s post-hoc test (**B**, **E**) or Mann-Whitney U test (**C**) or two-way ANOVA with Bonferroni’s multiple comparisons test (**F**). *P < 0.05.

Neither *Gsdmd*^*−/−*^ mice nor WT mice treated with an oxalate-rich diet manifested albuminuria (Supplementary Fig. S2C), suggesting interstitial or tubular cells are mainly involved in the mechanistic basis for the observed phenotypic differences. Furthermore, macrophages express high levels of Gsdmd (Fig. 1A) and infiltrate the kidney upon injury [25], and we observed greater interstitial macrophage infiltration in *Gsdmd*^*−/−*^ mice (Fig. 3E, F). Therefore, we concentrated on macrophages to assess their function and susceptibility to CaOx crystals in both *Gsdmd*^*−/−*^ and WT mice. We first conducted a series of tests encompassing assessments of phagocytic capacity, migratory ability, cell cycle dynamics, and their responsiveness to proinflammatory stimuli, using bone marrow-derived macrophages (BMDMs) from WT and *Gsdmd*^*−/−*^ mice. These evaluations showed no differences (Supplementary Fig. S6A-I). However, we observed significant differences between the two genotypes in macrophages regarding their susceptibility to CaOx crystals, but not in primary tubular cells. The number of SYTOX green-positive macrophages following exposure to CaOx crystals was significantly higher in *Gsdmd*^−/−^ BMDMs than in their WT counterparts (Fig. 8A, B). We obtained the same result when measuring lactate dehydrogenase (LDH) in the cell supernatant (Fig. 8C). However, we did not observe significant differences between *Gsdmd*^−/−^ and WT tubular cells in the SYTOX green assay or the LDH assay method (Supplementary Fig. S7A and S7B), indicating that more cytotoxicity of CaOx crystals on *Gsdmd*^−/−^ BMDMs, but no differences in tubular cells of two genotypes. Furthermore, immunofluorescent staining of pMLKL revealed greater induction of MLKL phosphorylation in *Gsdmd*^*−/−*^ BMDMs, indicating more necroptosis (Fig. 8D, E). Consistently, pretreatment with necroptosis inhibitors Nec1s or GSK872 reduced CaOx crystal-induced cytotoxicity in macrophages (Fig. 8F). These results suggest that the higher susceptibility of macrophages from *Gsdmd*^−/−^ mice to CaOx crystals leads to increased necroptosis, which might be associated with a delayed clearance of CaOx crystals in the kidney, potentially resulting in more severe nephrocalcinosis and profound kidney injury.

### Gene expression analysis revealed that gene sets related to leukocytes migration, mitochondrial function, and complement pathways are differentially expressed between *Gsdmd*^*−/−*^ and WT mice in chronic oxalate nephropathy

We also performed RNA sequencing on the kidneys of *Gsdmd*^*−/−*^ mice within this model. Cluster analysis clearly separated mice fed a control diet from those fed an oxalate-rich diet (Supplementary Fig. S8A and S8B). RNA sequencing analysis revealed significant changes in the gene sets related to leukocyte migration, chemokine pathways, mitochondrial dysfunction, and complement pathways between *Gsdmd*^*−/−*^ and WT mice (Supplementary Fig. S8C, S8D). These differences may be attributed to variations in the substances released during cell death. And these results confirm at the transcriptional level that oxalate nephropathy exacerbates in *Gsdmd*^*−/−*^ versus WT mice.

## Discussion

We had hypothesized that pyroptosis contributes to kidney injury in a chronic oxalate nephropathy model, and that interfering with GSDMD would ameliorate kidney injury. However, contrary to our expectations, Gsdmd deletion actually exacerbated kidney damage in this model, and we found that GSDMD acts as a modulator of necroptosis in addition to its well-established role as an executor of pyroptosis. Furthermore, pharmacological inhibition of necroptosis ameliorated kidney injury in *Gsdmd*^−/−^ mice within this model. Finally, our study revealed that *Gsdmd*^−/−^ macrophages were more prone to undergo necroptosis upon CaOx crystal stimulation compared to WT macrophages, potentially leading to the delayed clearance of CaOx crystals and more severe kidney injury.

Like pyroptosis, necroptosis is a form of regulated cell death, and previous studies have suggested its role in necroinflammation in kidney diseases [7, 12, 26, 27]. These cell death pathways are interconnected and share certain signaling molecules [28, 29]. Thus, modulating one form of regulated cell death may interfere with or promote other cell death pathways. For example, Yang et al. demonstrated that genetic deletion of Gsdmd led to an increase in necroptosis in a non-infectious liver injury model [30]. Furthermore, in such cases, targeting multiple cell death pathways may be beneficial in suppressing inflammation [8, 31]. In our experiment as well, the deletion of Gsdmd, a key player in pyroptosis, promoted necroptosis in the kidney in a chronic oxalate nephropathy model. Pharmacological inhibition of necroptosis was effective in alleviating kidney injury in *Gsdmd*^*−/−*^ mice. Our results imply that Gsdmd has a role as a direct or indirect regulator also of necroptosis. In other words, genetic deletion of Gsdmd may impact the expression or activation of necroptosis regulators involved in the posttranslational regulation of necroptosis initiators and executor such as RIPK1, RIPK3, and MLKL. For instance, caspase-8 is well known as an initiator of apoptosis, but it also inhibits necroptosis by cleaving RIPK1 [32]. Previous studies have shown that increased mitochondrial ROS leads to the inhibition of pro-caspase-8 cleavage, resulting in the upregulation of necroptosis, and the release of mitochondrial DNA activates RIPK1/RIPK3/MLKL necroptosis pathway [33–35]. Consistent with these reports, our RNA sequencing analysis revealed an upregulation of gene sets associated with mitochondrial dysfunction in *Gsdmd*^*−/−*^ mice.

Additionally, it has been reported that CaOx crystals are phagocytosed by macrophages, dissolved by phagolysosome, and subsequently eliminated [36]. Cathepsins, known as lysosomal components, are also involved in regulating necroptosis [2]. Based on these previous reports, we can also hypothesize that Gsdmd deletion might alter the function of lysosomal enzymes, including cathepsins, in macrophages. However, further investigations are needed to confirm these possibilities.

Apart from regulated cell death pathway, our study also revealed an increase in the expression of crystal-binding molecules CD44 and annexin II in primary renal tubular epithelial cells of *Gsdmd*^*−/−*^ mice. It is well-known that crystal adhesion to the surface of tubules is a crucial step in crystal growth and subsequent formation of tubular obstruction. Although we did not directly target these crystal-binding proteins, our findings suggest that the upregulation of these proteins, induced by Gsdmd deletion, might also contribute to the development of more severe kidney injury in chronic oxalate nephropathy.

Furthermore, beyond leukocyte-related genes, our RNA sequencing analysis revealed significant differences in gene expression associated with complement pathways and mitochondrial dysfunction in oxalate-fed WT and *Gsdmd*^*−/−*^ mice. These findings align closely with a previous report on human monocytes [37], suggesting that components associated with these gene sets may also be promising therapeutic targets for treating chronic oxalate nephropathy.

Our study has several limitations. Although we focused on GSDMD in macrophages, the use of *Gsdmd*^*−/−*^ mice does not fully rule out the possibility that GSDMD may also contribute to the overall phenotype in other cell types. Employing macrophage-specific *Gsdmd* knockout mice would help to address this aspect. Additionally, we did not investigate the molecular mechanisms underlying the susceptibility of BMDMs to necroptosis in this study. Further molecular investigations into our findings are still needed.

Our study underscores the critical role of macrophages in chronic oxalate nephropathy and highlights that molecules involved in regulated cell death often share signaling pathways, allowing these cell death processes to influence one another. These insights offer new directions for research into the regulation of cell death, particularly necroptosis.

### Data sharing statement

The transcriptome data have been deposited and are available in the Gene Expression Omnibus (GEO) database <https://www.ncbi.nlm.nih.gov/geo/> (Accession number: GSE279923).

## Supplementary information

Supplementary information

original data

original data

original data
