# Supplementary material for: Gasdermin D deficiency aggravates nephrocalcinosis-related chronic kidney disease with rendering macrophages vulnerable to necroptosis
Source: Cell Death Dis. 2025 Apr 13;16(1):283. doi: 10.1038/s41419-025-07620-1 (PMC11993636; doi:10.1038/s41419-025-07620-1)
Supplement: Supplementary file 1 — Supplementary information [file 41419_2025_7620_MOESM1_ESM.docx]

**Supplementary Information for**

**Gasdermin D drives deficiency aggravates nephrocalcinosis-related chronic kidney disease with rendering macrophages vulnerable to necroptosis**

Yoshihiro Kusunoki^1,2^, Chenyu Li^1,3^, Hao Long^1,4^, Kanako Watanabe-Kusunoki^1,2^, Meisi Kuang^1^, Julian A. Marschner^5^, Andreas Linkermann^6,7,8,9^, Stefanie Steiger^1^, Hans-Joachim Anders^1^

1 Renal Division, Department of Medicine IV, Ludwig Maximilian University Hospital, Ludwig Maximilian University, Munich, Germany.

2 Department of Rheumatology, Endocrinology, and Nephrology, Faculty of Medicine and Graduate School of Medicine, Hokkaido University, Sapporo, Japan

3 Department of Medicine, Renal Electrolyte and Hypertension Division, Perelman School of Medicine, University of Pennsylvania, Philadelphia, PA, USA

4 Department of Urology, The Affiliated Hospital of Southwest Medical University, Luzhou, China

5 Department of Pharmacy, Ludwig-Maximilians-University, Munich, Germany

6 Department of Medicine V, University Medical Centre Mannheim, University of Heidelberg, Mannheim, Germany.

7 Division of Nephrology, Medical Clinic III, University Hospital Dresden, Technische Universität Dresden, Dresden, Germany.

8 Department of Internal Medicine 3, University Hospital Carl Gustav Carus at the Technische Universität Dresden, Dresden, Germany.

9 Division of Nephrology, Department of Medicine, Albert Einstein College of Medicine, Bronx, NY, USA.

**Table of contents**

**Supplementary methods**

Single nuclei RNA sequencing 1

Bulk RNA sequencing 1

Histological analysis 1

RNA Preparation and Real-Time Quantitative PCR 2

Isolation of bone marrow cell and differentiation of macrophage 2

Measurement of urinary creatinine, albumin, calcium, oxalic acid, and blood urea nitrogen 2

Preparation of necrotic cell supernatant 3

Migration assay 3

Scratch assay 3

Phagocytosis assay 3

Cell cycle analysis and Cell count 3

Polarization and characterization of macrophages 4

Isolation of primary renal tubular cells (pTECs) from murine kidney 4

Cell death detection assay for BMDMs and pTECs 4

BMDMs and pTECs stimulation for Inflammasome assays 5

**Supplementary Tables**

Supplementary table S1. Upregulated pyroptosis-related genes in kidney of *Gsdmd^-/-^* mice fed an

oxalate-rich diet 6

Supplementary table S2. Murine primer sequences 6

**Supplementary Figures**

Supplementary figure S1. 7

Supplementary figure S2. 8

Supplementary figure S3. 9

Supplementary figure S4. 10

Supplementary figure S5. 11

Supplementary figure S6. 12

Supplementary figure S7. 14

Supplementary figure S8. 15

**Supplementary References** 16

**Supplementary Methods**

**Single nuclei RNA sequencing**

Raw data or count matrices were obtained from GSE139107 [1]. The single nuclei RNA-seq data were processed using the Scanpy pipeline [2], which enabled the integration and merging of different batches of data. Quality control was performed by utilizing the threshold values as reported in the original article. Data normalization was conducted using the scran package [3], which included assuming equal size factors, normalizing library sizes to counts per million, and log-transforming the count data. Principal component analysis was performed based on the 3 000 highly variable genes. To eliminate technical differences and preserve biological differences, the harmony integration pipeline [4] was employed to reduce data dimensions and remove batch effects. Uniform Manifold Approximation and Projection (UMAP) was applied for unsupervised clustering based on the first 50 integrated principal components with maxiter=30, min_dist=1 and spread=2. Gene enrichment scores were calculated using "scanpy.tl.score_genes()" functions [5]. Additionally, pseudo-temporal analysis was conducted using PAGA (Partition-based Graph Abstraction)[6] to infer developmental trajectories and cellular differentiation pathways for the kidney cell subset.

**Bulk RNA sequencing**

Total RNA was extracted from kidney samples of *Gsdmd^-/-^* and WT mice, both on a normal diet and an oxalate-rich diet. For the in vitro experiment, total RNA was isolated from pTECs of *Gsdmd^-/-^* and WT mice. The quality of RNA was assessed by analyzing the RNA Integrity Number (RIN) using a Bioanalyzer 2100 instrument (Agilent, Santa Clara, CA). RNA samples with a RIN value > 8 were selected for RNA sequencing. Library preparation and sequencing were conducted by Beijing Genomics Institute (BGI) using the DNBSEQ (G400) platform, and the RNA samples underwent 100-base-pair paired-end sequencing. The bioinformatics workflow, which included data filtering, mapped transcript prediction, differential gene expression analysis and gene ontology, was performed using the protocols of HISAT2 [7], samtools [8], FeatureCounts [9]/eisaR [10], and clusterProfiler [11]. The complete dataset is available in the Gene Expression Omnibus (GEO) under accession number GSE279923.

**Histological analysis**

Kidney tissues were embedded in paraffin and sliced into 2 μm. Tubular injury was assessed using the Periodic acid-Schiff (PAS) reagent, and the degree of injury in ten images using a ×20 objective was scored based on the percentage of tubules displaying tubular cell necrosis, dilation, vacuolization, and infiltration of inflammatory cells, using the scoring system as follows: 0 = negative, 1 = 1-20%, 2 = 21-40%, 3 = 41-60%, 4 = 61-80%, 5 = 81-100%. Pizzolato’s staining visualized CaOx crystal deposition, which in ten images using a x20 objective was quantified (% area) with Image J software (National Institutes of health, Bethesda, MD) as described previously [12]. Fibrotic areas were identified by Sirius red staining, and quantification in ten images using a x20 objective was done utilizing Image J software. F4/80+ macrophages (NB600-404, NOVUS Biologicals, Littleton, CO, USA) were identified by immunostaining and analyzed by assessing the positively stained area in ten images using a ×20 objective. CD44 and Annexin II were identified by immunostaining for CD44 and Annexin II (both Abcam, Cambridge, United Kingdom) and positive areas in ten images using a x20 objective. Quantification of the immunostaining was assessed using Image J software. Immunohistochemistry was performed following endogenous peroxidase inhibition and heat-induced antigen retrieval. The sections were then blocked and incubated with primary antibodies including rabbit anti-Gasdermin D (ab219800, Abcam), Rabbit anti-pMLKL (ab196436, Abcam,) and Rabbit anti-mouse Cleaved caspase-3 (#9661, Cell Signaling Technology, Danvers, MA, USA). Diaminobenzidine was used for detection and images were captured using a Leica DMRBE microscope (Leica Microsystems, Wetzlar, Germany). pMLKL-positive area was quantified (% area) in ten images using a x20 objective, and Cleaved caspase-3 positive cells were counted in ten images using a x20 objective. TdT-mediated dUTP-biotin nick end labeling (TUNEL)-positive kidney cell death (11684795910, Roche, Mannheim, Germany) in ten x20 objective images was counted. An observer blinded to the experimental condition performed all assessments.

**RNA Preparation and Real-Time Quantitative PCR**

Total RNA was isolated from kidneys using an RNA extraction kit (12183018, Invitrogen, Carlsbad, CA, USA) following the manufacturer’s instructions. RNA quality was assessed using agarose gels before transcription into cDNA using reverse transciptase (Superscript II; 18064022, Invitrogen). Real-time quantitative PCR was performed using SYBRGreen PCR master mix, analyzed with Light Cycler 480 (Roche, Mannheim, Germany). All gene expression values were normalized using 18s rRNA as a house keeping gene. All primers used for amplification were from Metabion (Martinsried, Germany) and are listed in supplementary table 1.

**Measurement of urinary creatinine, albumin, calcium, oxalic acid, and blood urea nitrogen (BUN)**

Urine samples were collected on day 0 and day 20. Urinary creatinine (DiaSys Diagnostic Systems, Holzheim, Germany), urinary albumin (E90-134, Bethyl Laboratories, Montgomery, MD), urinary oxalic acid (MAK179, Siga Aldrich, USA), urinary calcium (MAK022, Sigma Aldrich), and serum BUN (EIABUN, Thermo Fisher Scientific, USA) were measured using commercially available kits. All ELISA and colorimetric assays were performed according to the manufacturer's protocols.

**Isolation of bone marrow cell and differentiation of macrophage**

WT and *Gsdmd^-/-^* mice were sacrificed by cervical dislocation, and femur and tibia bones were isolated. Then, the bones were immersed in 75 % ethanol for 1 min before being stored in PBS. The epiphyses of the bones were removed and a 25-gauge needle was inserted into the bone cavity, and bone marrow cells were harvested by flushing with PBS. Harvested cells were incubated in 0.155 M NH4Cl lysis buffer for 2 min and filtered with 70 μm cell strainer. Then, cells were centrifuged at 300 x g for 5 min and resuspended in L929 conditioned medium (LCCM). The cells were incubated for 7 days and used as bone marrow-derived macrophages (BMDMs).

**Preparation of necrotic cell supernatant**

Ten million primary murine tubular cells were collected in 1 ml of PBS and subjected to 5 cycles of freezing in liquid nitrogen followed by thawing in a water bath. The cells were then centrifuged at 500 x g for 5 min, and the supernatant was collected as the necrotic lysate ‘necrotic soup: NTS’. This necrotic lysate was subsequently utilized in specific assays to assess macrophage responses at a concentration equivalent to that derived from the same number of pTECs.

**Migration assay**

BMDMs were serum-starved by removing FBS for 3 h. Cells were collected, and 2.0 x 10^5^ BMDMs in 200 μ l of DMEM supplemented with 0.1 % FBS were seeded into each Transwell insert (3464, Corning). For the bottom wells, 500 μl of culture medium (either DMEM supplemented with 0.1 % FBS or DMEM supplemented with 0.1 % FBS and NTS) was added. After 5 h of incubation, the cells were fixed with 4 % PFA and stained with 0.1 % crystal violet. Cells remaining on upper surface of the membrane were removed by scrapping with a cotton swab, and images were captured using a Nikon Eclipse Ti2 microscope (NIKON, Tokyo, Japan).

**Scratch Assay**

2.5 x 10^5^ BMDMs in 500 μl LCCM were seeded in 24-well plates and incubated overnight. Scratches were made using pipette tips, and each well was washed twice with PBS. Cells were then incubated with or without indicated reagents in LCCM. Images were captured every 2 h using a Nikon Eclipse Ti2 microscope (NIKON, Tokyo, Japan).

**Phagocytosis assay**

5.0 x 10^5^ BMDMs were seeded into 12-well plates and incubated overnight in LCCM with the indicated reagents. Phagocytosis assay was performed using a Phagocytosis Assay Kit (Cay 50029, Cayman Chemical, Ann Arbor, Michigan, USA), following the manufacturer’s instructions. Briefly, after removing the LCCM, 500 μl of a 1:250 dilution of IgG-coated latex beads in fresh LCCM were added to each well and incubated for 3 h. The wells were washed twice with PBS, and the cells were collected. Flow cytometry was performed using a FACSCalibur (Becton Dickinson, New Jersey, USA), and data were analyzed with FlowJo version 10 software (Tree Star, Ashland, OR, USA).

**Cell cycle analysis and cell count**

For the comparison of proliferation between WT and *Gsdmd^-/-^* macrophages, cell cycle analysis and cell counting were performed. For cell cycle analysis, 5.0 x10^5^ cells were collected and washed with PBS. The cells were resuspended in 300 μl of PBS, and 700 μl of 100 % ethanol were added dropwise to the pellet while vortexing. The BMDMs were fixed for 30 min at 4 °C. Cells were then washed with PBS and centrifuged at 850 x g for 5 min, followed by resuspension in 300 μl of RNase (EN0531, Thermo Fisher Scientific, Waltham, MA, USA). The cells were incubated with PI solution (421301, BioLegend, Fell, Germany) for 30 min at room temperature. Flow cytometry data were acquired on a FACSCalibur (Becton Dickinson) and analyzed using FlowJo version 10 software (Tree Star).

For cell counting, 1.0 x 10^5^ BMDMs were seeded into FACS tubes and 12-well plates (1.0 x 10^5^cells/ well) with LCCM. Baseline cell counts were determined using flow cytometry on a FACSCalibur. After 48 h of incubation, cells in the 12-well plates were collected, and cell numbers were enumerated via FACSCalibur (Becton Dickinson), and analyzed with FlowJo version 10 software (Tree Star). AccuCheck counting beads (PCB100, Thermo Fisher Scientific) were used to determine the absolute cell counts following the manufacturer’s instructions.

**Polarization and characterization of macrophages**

BMDMs were seeded in 12-well plates and incubated overnight in LCCM with indicated reagents.

After incubation, cells were stained with FITC anti-mouse CD80 antibody (104705, BioLegend) and APC anti-mouse CD192 (CCR2) antibody (150627, BioLegend).

M1-like macrophages were defined as the expression of both CD80 and CCR2, and quantified via Flow cytometry on a FACSCalibur (Becton Dickinson). The data were analyzed with FlowJo version 10 software (Tree Star).

To assess pro-inflammatory characteristics with enzyme-linked immunosorbent assay (ELISA), cell supernatants were collected and centrifuged to remove dead cells. The supernatants were analyzed using mouse IL-6 and TNFα-specific ELISA kits (88-7324-22, 88-7064-22, Fisher Scientific, Germany, respectively) according to manufacturer’s instructions.

**Isolation of primary renal tubular cells (pTECs) from murine kidney**

pTECs were isolated as previously described [13, 14]. In brief, kidneys from 3 to 4-week-old mice were homogenized and incubated with collagenase D (1.5 mg/mL) for 30 min at 37 °C. The digested tissue was passed through 20- and 25-gauge needles, and filtered through a 70 μm strainer. Red blood cells were lysed using 0.155 M NH4Cl lysis buffer. The cell suspension was layered onto 31 % Percoll solution and then centrifuged at 1600 x g for 15 min at 4 °C. The cells were resuspended and cultured with predefined K1 medium.

**Cell death detection assay for BMDMs and pTECs**

BMDMs and pTECs were seeded into 96-well plates in LCCM at densities of 5.0 x 10^4^ cells/ well and 1.0 x 10^4^ cells/ well, respectively, and incubated overnight. Cells were treated with CaOx with or without preincubation of the indicated reagents. To assess the cytotoxicity of CaOx crystals, cells were incubated with 1mg/ml CaOx solution for 24 h. Preincubation with Nec-1s or GSK872 for 1 h was conducted to evaluate their inhibitory effects on CaOx-induced cytotoxicity. For cytotoxicity assessment, cell-free supernatants were collected at 24 h, and lactate dehydrogenase (LDH) release was measured using LDH assay kit (11644793001, Roche, Mannheim, Germany) according to the manufacturer’s instructions.

For SYTOX green (SG) stainings, BMDMs and pTECs were seeded into 96-well plates (1.25 x 10^4^ cells/ well) and incubated with 1 mg/ml CaOx solution for 4 h. The cells were stained with SG (0.5 μM, BioLegend), and images were obtained using a Nikon Eclipse Ti2 microscope (NIKON, Tokyo, Japan). The SG-positive cells in each well were defined as dead cells, and quantified using NIS-Elements Viewer software (NIKON).

**BMDMs and pTECs stimulation for inflammasome assays**

To assess inflammasome activation, BMDMs or pTECs were seeded in 96-well plates at densities of 5.0 x 10^4^ cells/well and 1.0 x 10^5^ cells/well, respectively. After overnight incubation, the culture media were replaced with fresh LCCM for BMDMs and fresh K1 medium for pTECs. The cells were then treated with 50 μg/ml nigericin following 3 h-preincubation of 1 μg/ml LPS for priming. After the incubation, cell-free supernatants were harvested at different time points (0.5 h, 1 h, 3 h, and 24 h) for IL-1β enzyme-linked immunosorbent assay (ELISA) (Invitrogen) and LDH assay (Roche).

**Supplementary tables**

**Supplementary table S1. Upregulated pyroptosis-related genes in kidney of *Gsdmd^-/-^* mice fed an oxalate-rich diet**

| **Gene name** | **Log2 fold change** | **Adjusted P value** |
| --- | --- | --- |
| Casp1 | 1.839 | 8.528E-6 |
| Il-1b | 2.105 | 2.811E-4 |
| Nlrp3 | 1.286 | 3.331E-4 |
| Gsdmd | 1.180 | 1.592E-6 |

**Supplementary table S2. Murine primer sequences**

| **Target** | **Primer sequences** |
| --- | --- |
| 18s | Forward: 5’-GCAATTATTCCCCATGAA-3’ |
|  | Reverse: 5’-AGGGCCTCACTAAACCAT-3’ |
| KIM-1 | Forward: 5’-TGGTTGCCTTCCGTGTCT-3’ |
|  | Reverse: 5’-TCAGCTCGGGAATGCACA-3’ |
| TNFα | Forward: 5’-AGGGTCTGGGCCATAGAACT-3’ |
|  | Reverse: 5’-CCACCACGCTCTTCTGTCTA-3’ |
| TGFβ1 | Forward:5’-CAACCCAGGTCCTTCCTAAA-3’ |
|  | Reverse: 5’-GGAGAGCCCTGGATACCAAC-3’ |
| CD44 | Forward: 5’-AGCGGCAGGTTACATTCAAA-3’ |
|  | Reverse: 5’-CAAGTTTTGGTGGCACACAG-3’ |
| Annexin II | Forward: 5’-GCACATTGCTGCGGTTTGTCAG-3’ |
|  | Reverse: 5’-CACCAACTTCGATGCTGAGAGG-3’ |

**Supplementary figures**

**
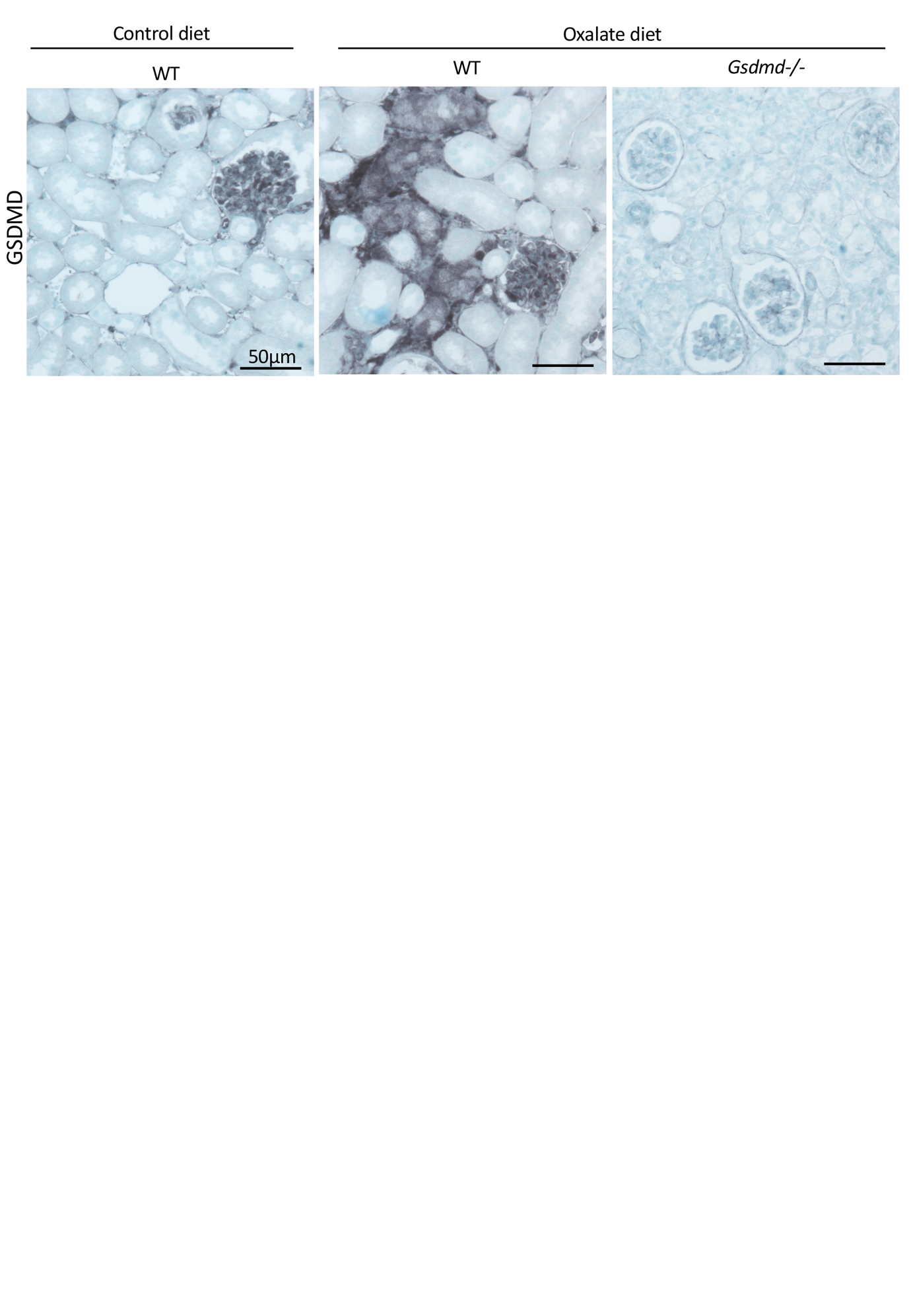
**

**Supplementary figure S1.** Representative high-magnification images of Gsdmd staining in the kidneys of WT mice fed a control diet, and of WT and *Gsdmd^-/-^* mice fed an oxalate-rich diet.

**
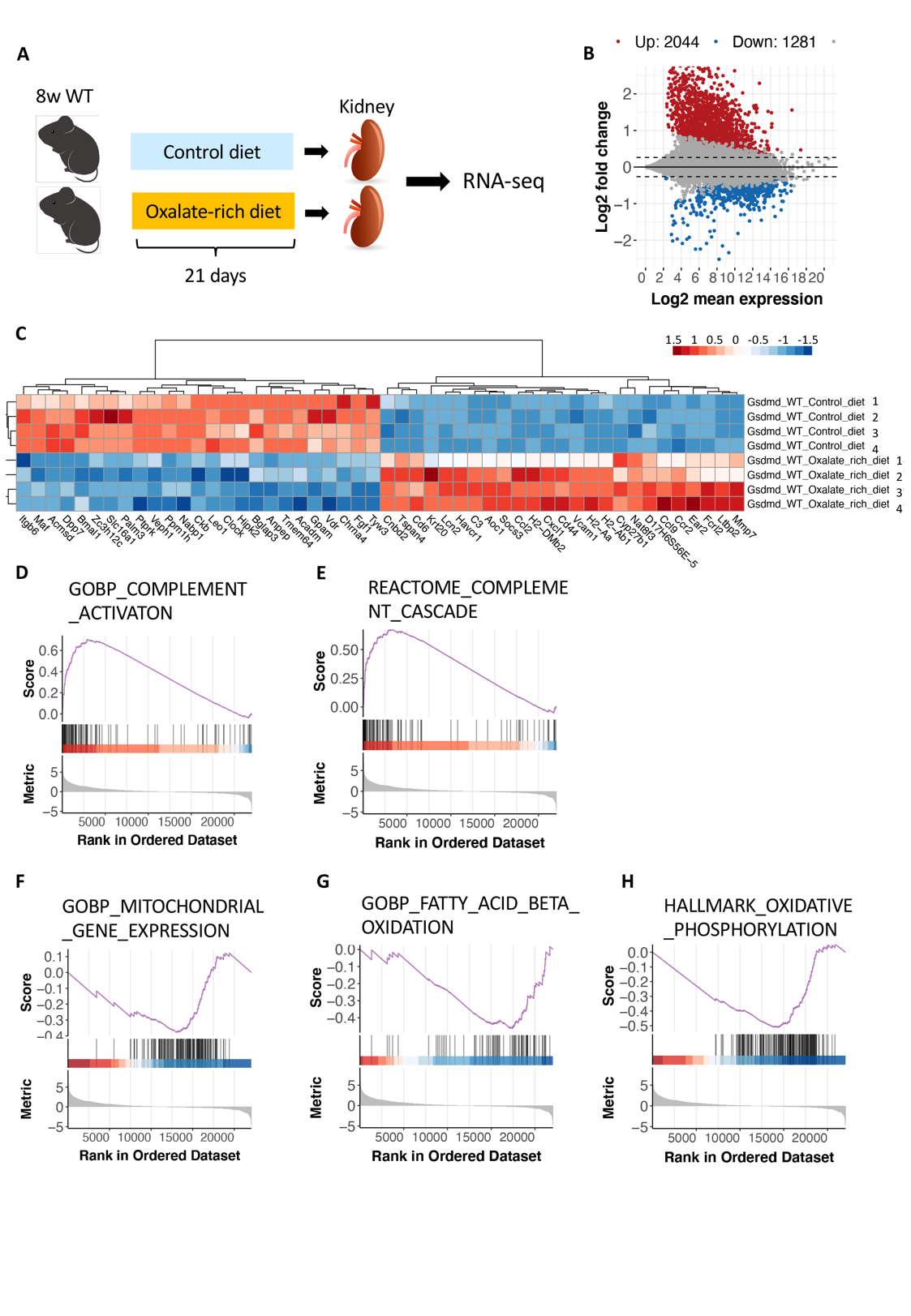
**

**Supplementary figure S2. Bulk RNA sequencing analysis revealed that gene sets related to leukocytes migration, mitochondrial function, and complement pathways are upregulated in a chronic oxalate nephropathy mice model.** (A) Illustration of the study design. Eight-week-old C57BL/6N mice received control diet or oxalate rich diet for 21 days. The mice were sacrificed on day 21 and analyzed for RNA sequencing. (n = 4 mice/group) (B) An MA plot illustrating the shrink log2 fold change between the kidneys of WT mice fed a control-diet and those fed an oxalate-rich diet. (C) A heatmap presenting the biological replicates of the top differentially expressed genes between the kidneys of WT mice fed a control diet and those fed an oxalate-rich diet. The heatmap and dendrogram display the z-scores of normalized counts for these genes. (D-H) Representative enrichment plots from GSEA analysis based on the gene enrichment profiles of WT mice fed an oxalate-rich diet versus those fed a control diet. The plots highlight the enrichment of transcriptional signatures related to complement pathways and mitochondrial function.

**
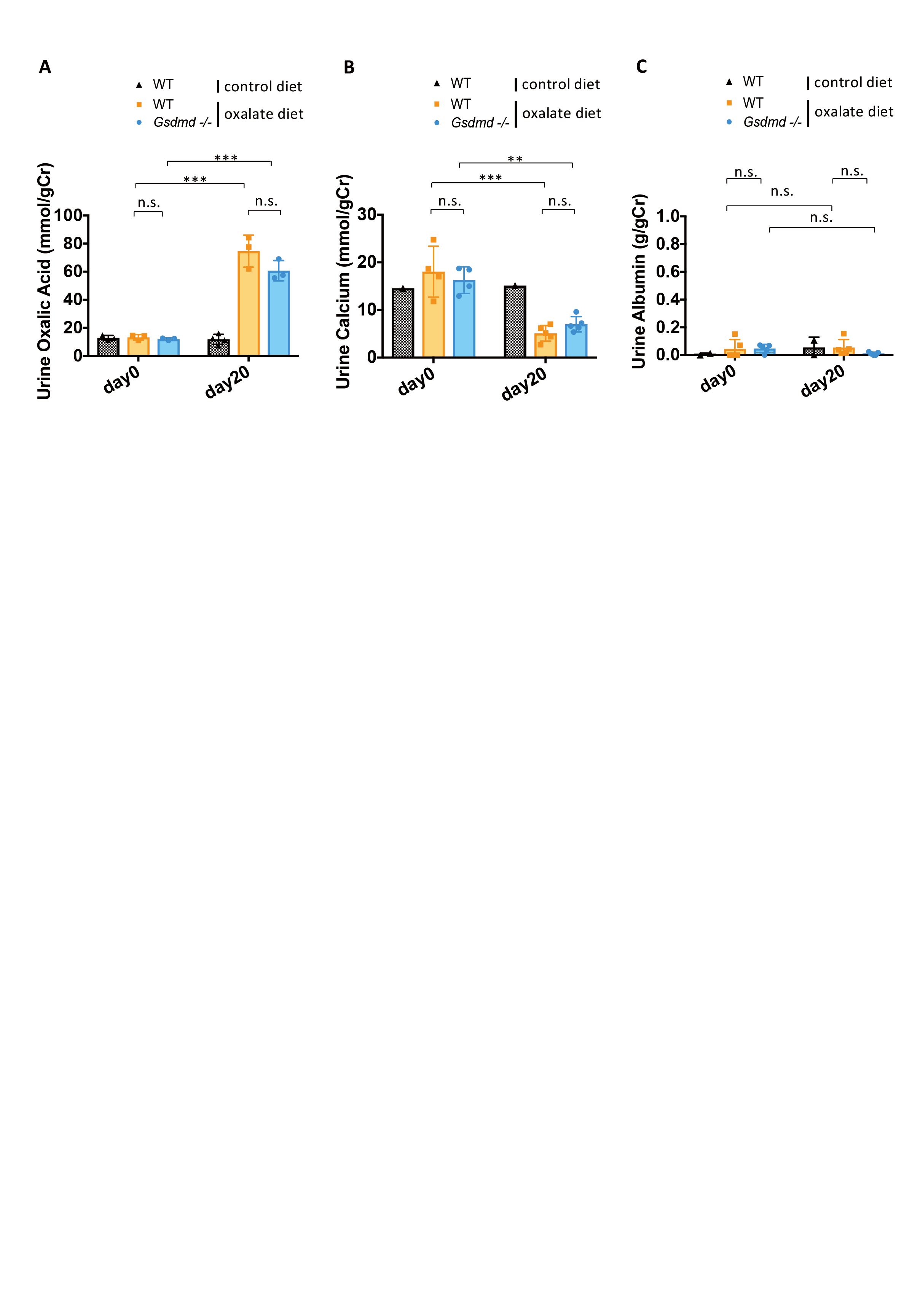
**

**Supplementary figure S3. Urinary analysis results consistent with CaOx crystal-induced chronic nephropathy. (A)** Urinary concentrations of oxalic acid were measured at baseline and on day 20 in WT mice fed a control diet, and in WT and *Gsdmd^-/-^* mice fed an oxalate-rich diet. **(B)** Urinary concentration of calcium at baseline and on day 20 in WT mice fed a control diet, and in WT and *Gsdmd^-/-^* mice fed an oxalate-rich diet. (C) Urine albumin-to-creatinine ratio (ACR) at baseline and on day 20 in WT mice fed a control diet, and in WT and *Gsdmd^-/-^* mice fed an oxalate-rich diet. Data are presented as mean ± SD. P values are calculated using two-way ANOVA with Bonferroni’s multiple comparisons test. n.s., not significant; **P < 0.01, ***P < 0.001

**
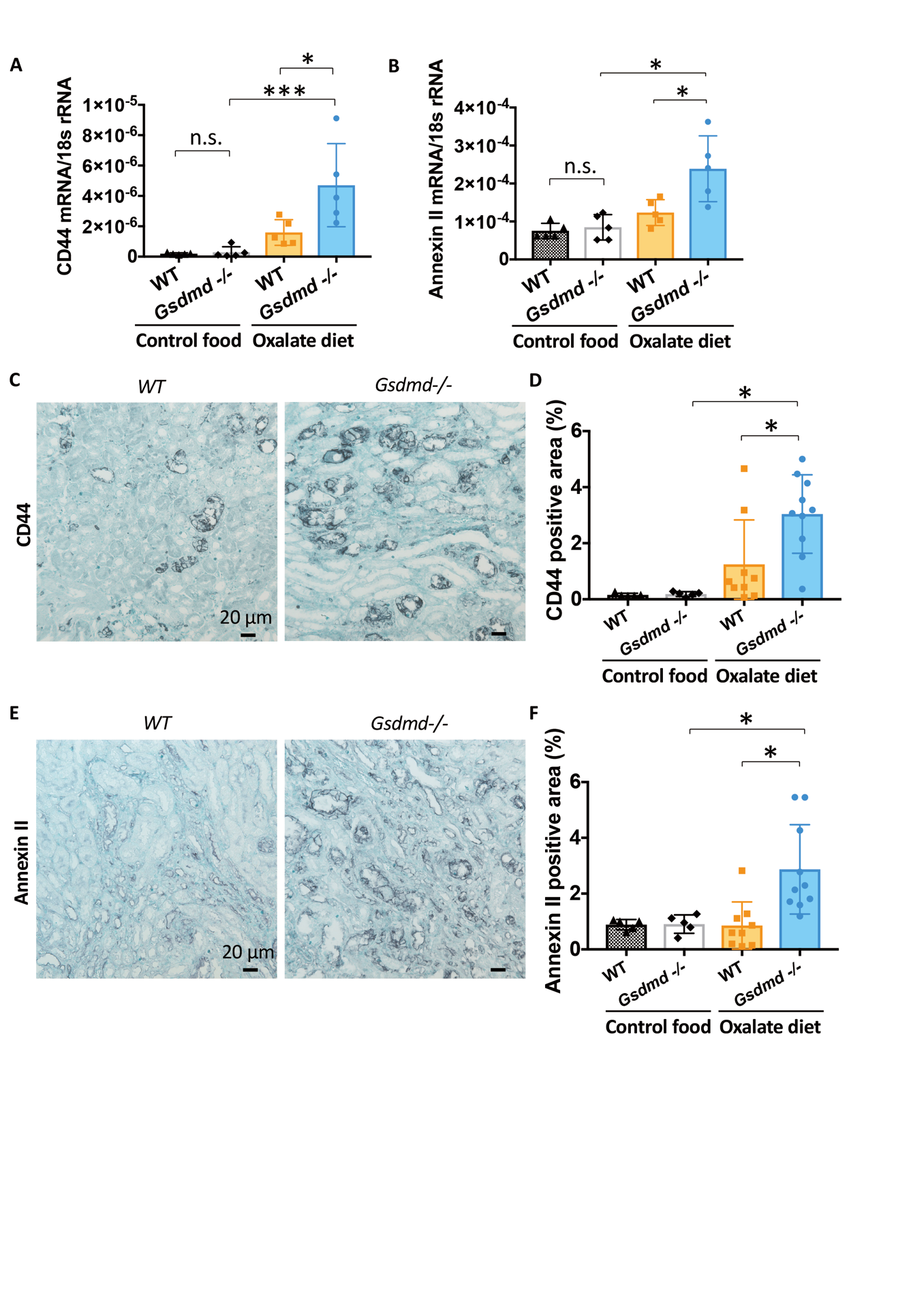
**

**Supplementary figure S4. The expression of crystal-binding molecules CD44 and Annexin II escalated in the kidneys of *Gsdmd^-/-^* mice compared to their WT counterparts.** (A) mRNA expression of CD44 in kidney RNA isolates from WT and *Gsdmd^-/-^* mice fed an oxalated-rich diet. (B) mRNA expression of Annexin II in kidney RNA isolates from WT and *Gsdmd^-/-^* mice fed an oxalated-rich diet. (C) Representative images of CD44 staining in the kidneys of WT and *Gsdmd^-/-^* mice fed an oxalated-rich diet. (D) Quantification of CD44-positive areas. (E) Representative images of Annexin II staining in the kidneys of WT and *Gsdmd^-/-^* mice treated with an oxalated-rich diet. (F) Quantification of Annexin II-positive areas. Data are presented as mean ± SD. P values are calculated using one-way ANOVA with Turkey’s post-hoc test. n.s., not significant; *P < 0.05, ***P < 0.001


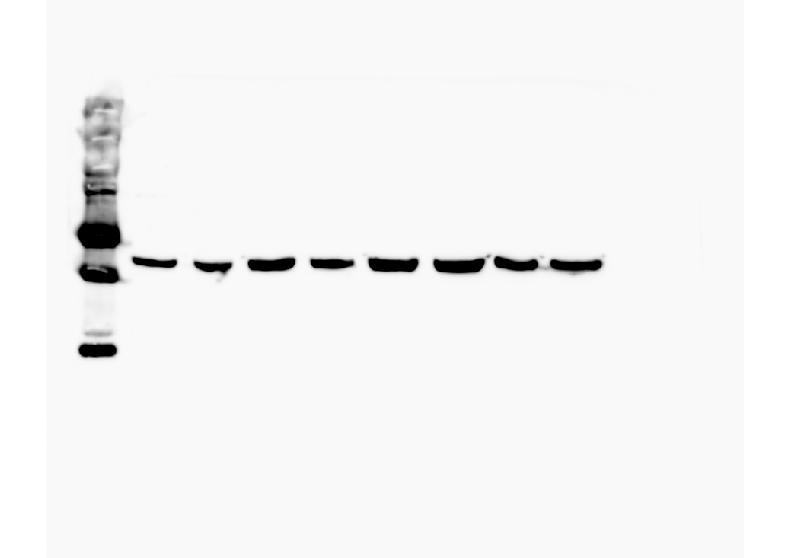


β actin

RIPK3

MLKL


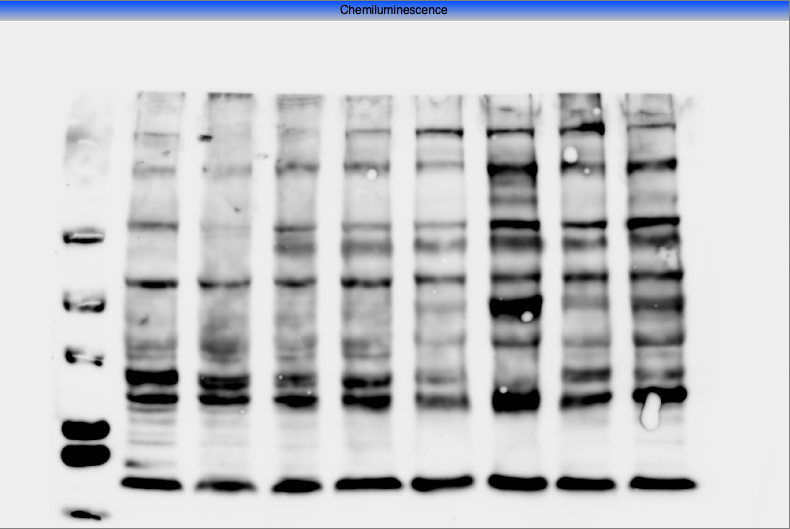

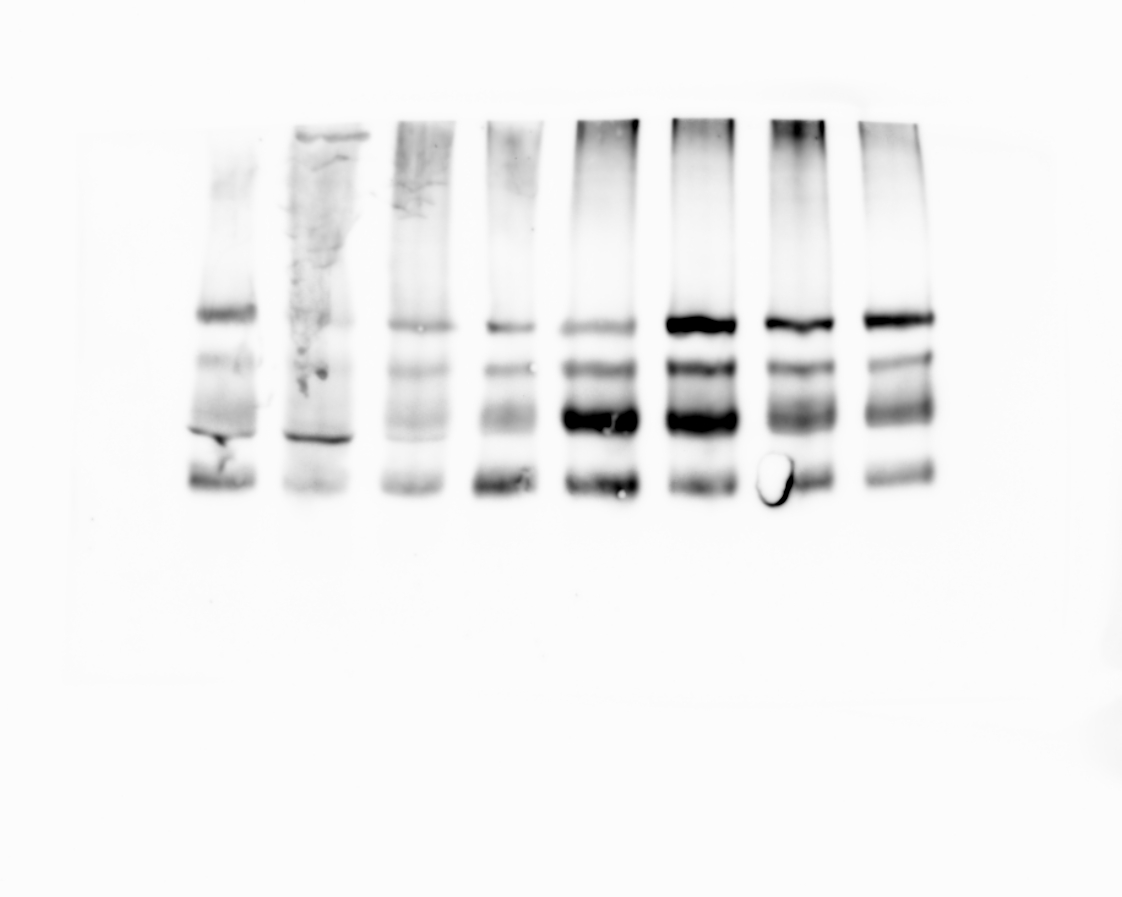

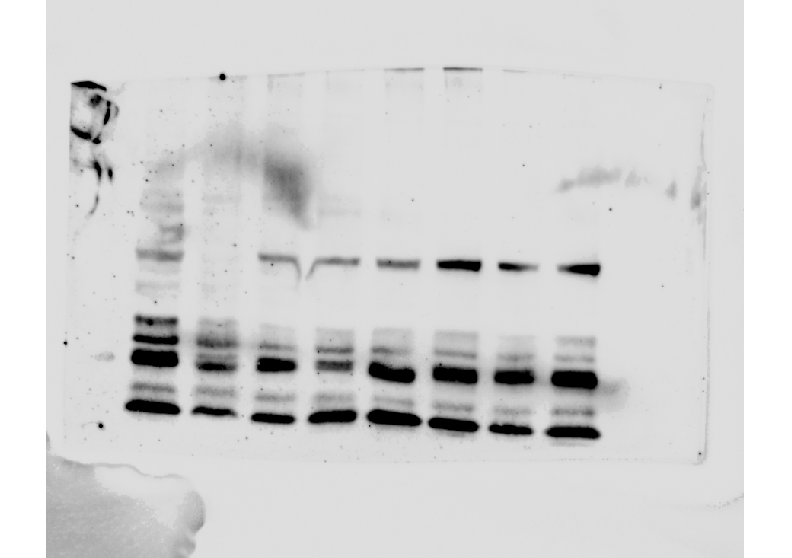


RIPK1

Oxalate diet

Control diet

*Gsdmd -/-*

*WT*

*Gsdmd -/-*

**kDa**

*WT*

- 42

- 57

- 54

- 78

**Supplementary figure S5.**

Immunoblot analysis of RIPK1, RIPK3, and MLKL in the kidneys of oxalate-rich diet treated WT and *Gsdmd^-/-^* mice. β-actin was used as a loading control.

**
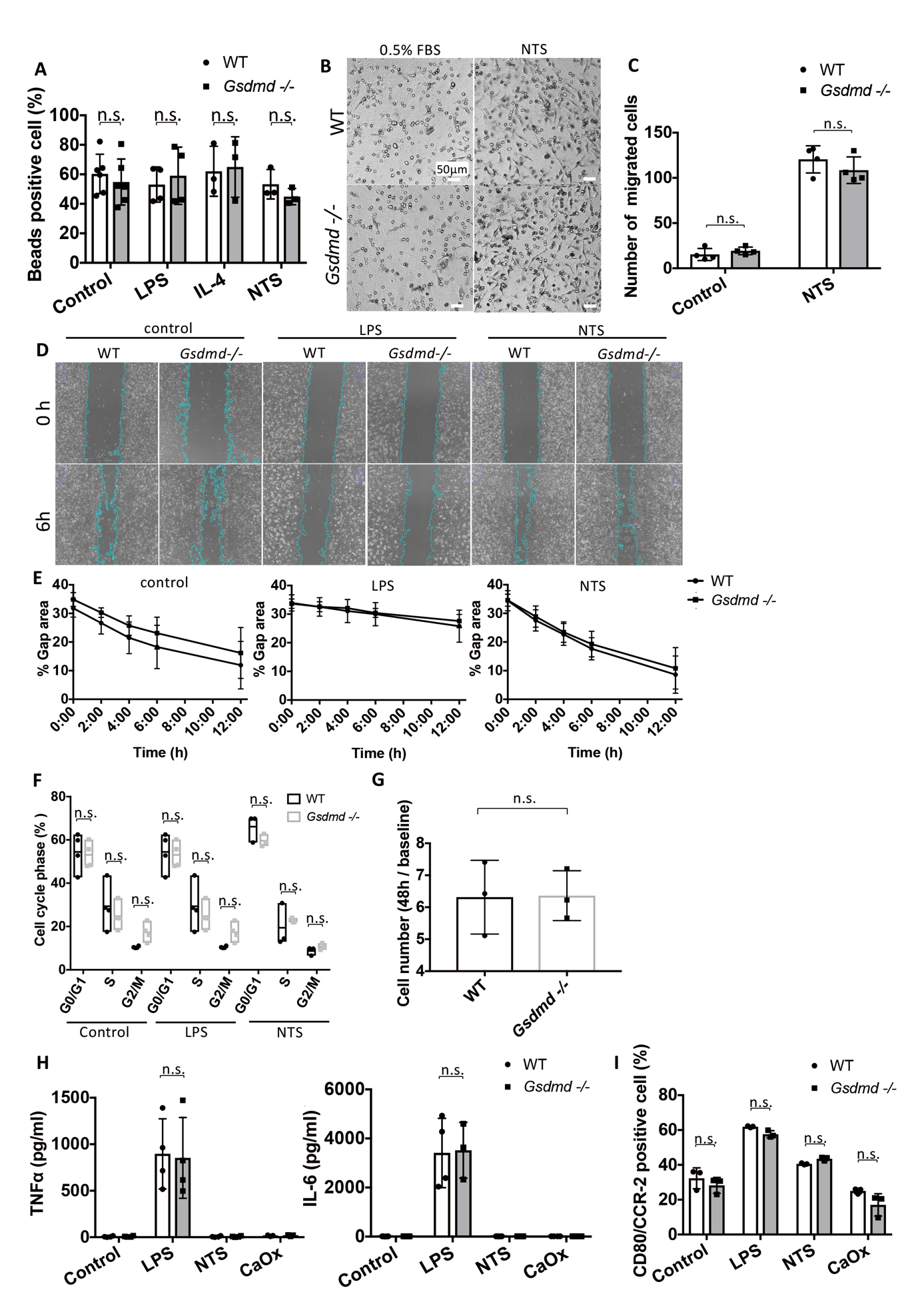
**

**Supplementary figure S6. No significant differences were observed in the functional and proliferative abilities, as well as inflammatory responses, between BMDMs from WT and *Gsdmd^-/-^* mice.** (A) Phagocytosis ability of BMDMs from WT mice and *Gsdmd^-/-^* mice. (B) Representative brightfield images of cell migration 6 h after incubation with 0.5% FCS or necrotic soup [15], assessed by the Transwell assay. (C) Quantification of migrated BMDMs. (D) Representative bright field images showing cell migration at 0 and 6 h after treatment with LPS or necrotic soup [15] in the scratch assay. (E) Quantification of gap areas, which indicates migration of BMDMs in in the scratch assay. (F) Distribution of cell cycle phase in BMDMs 24 h after treatment with LPS or NTS. (G) Ratio of the number of BMDMs counted at baseline and 48 h, as assessed by flow cytometry. (H) Concentrations of TNFα and IL-6 in cell-free supernatants 24 h after treatment with LPS, NTS, or CaOx crystals, measuring using ELISA. (I) Percentage of M1 marker-positive macrophages (CD80+ CCR2+) after stimulation with LPS, NTS, and CaOx crystals, as determined by flow cytometry. Data are presented as mean ± SE from at least 3 independent experiments. P values are calculated using Mann-Whitney U test. n.s., not significant


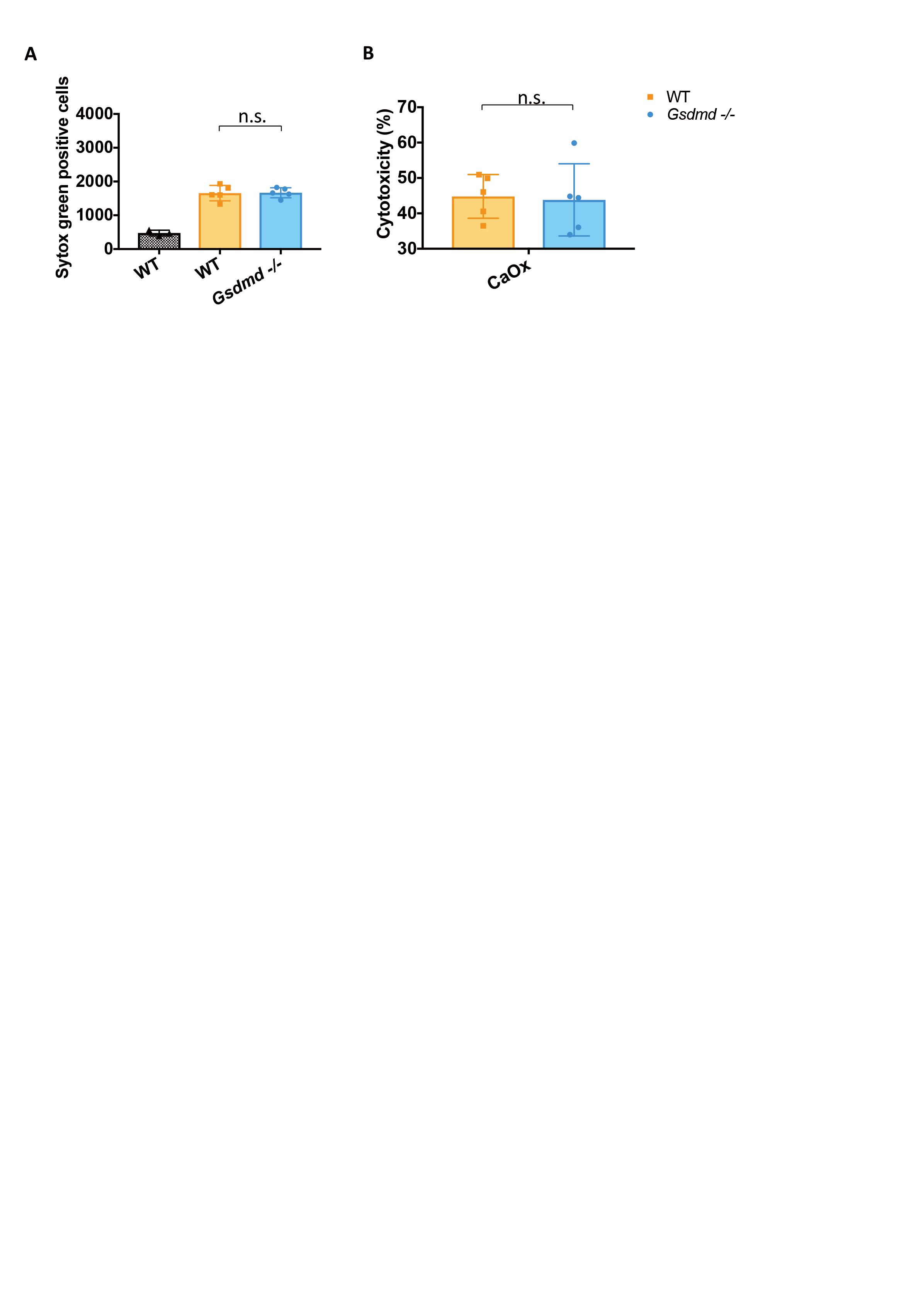


**Supplementary figure S7. The extent of CaOx-induced cell injury was comparable between Gsdmd-/- and WT mouse tubular cells.** (A) Quantification of SG-positive cells at 4 h after stimulation with CaOx crystals. (B) TECs were stimulated with 1 000 μg/ml CaOx crystals for 18 h, and cell-free supernatants were collected for LDH assay. Data are presented as mean ± SE from at least 3 independent experiments. P values are calculated using one-way ANOVA with Turkey’s post-hoc test (A) or Mann-Whitney U test (B). n.s., not significant

**
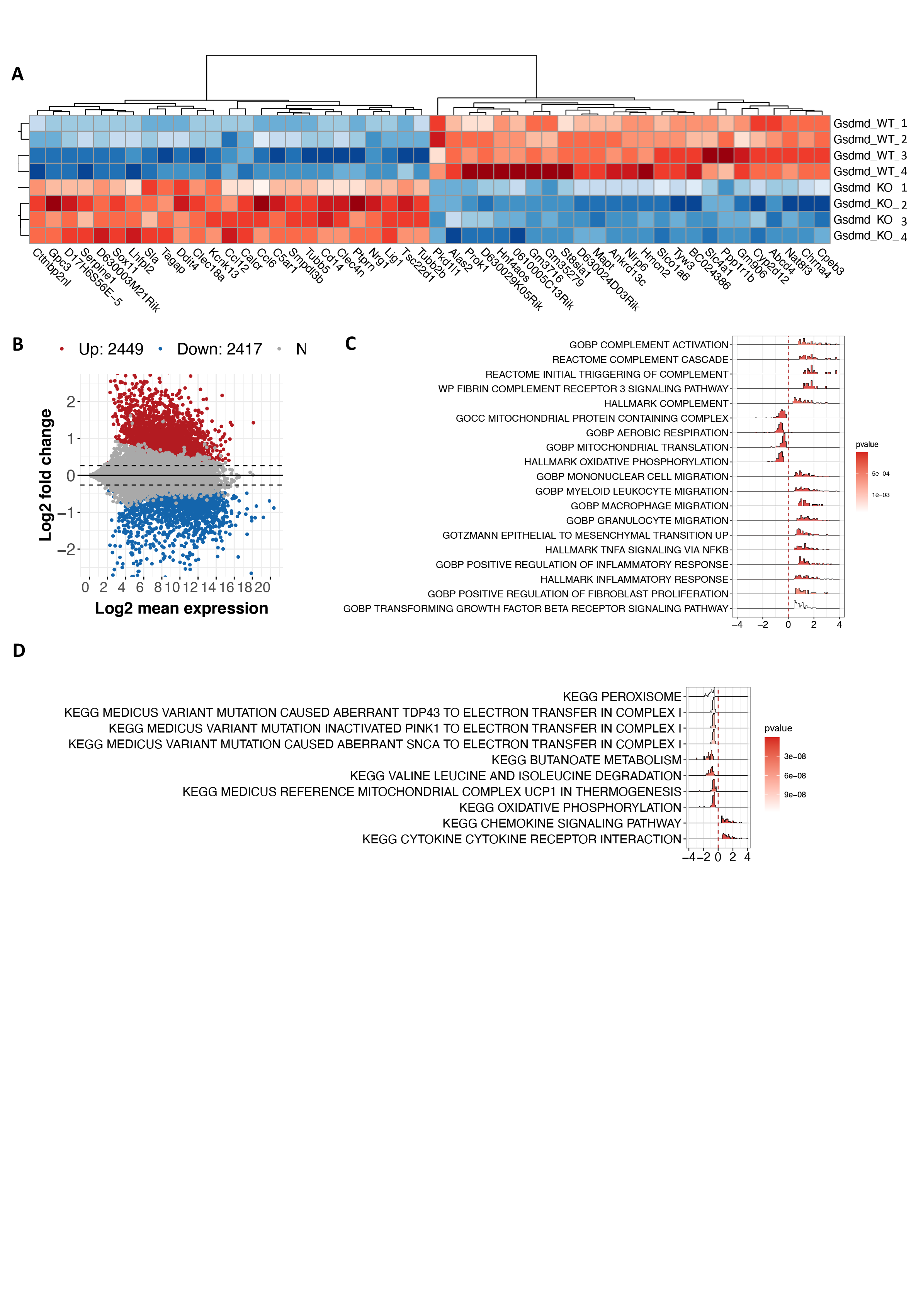
**

**Supplementary figure S8. Comprehensive gene expression analysis revealed that gene sets related to leukocytes migration, mitochondrial function, and complement pathways are differentially expressed between *Gsdmd^-/-^* and WT mice in chronic oxalate nephropathy.** (A) An MA plot was created to display the shrink log2 fold change between the kidneys of *Gsdmd^-/-^* and WT mice fed an Oxalate-rich diet. Genes found to be significantly different in expression, as indicated by an adjusted p-value, are labeled either in red or blue. (B) A heatmap presenting the biological replicates of the top differentially expressed genes between the kidneys of *Gsdmd^-/-^* and WT mice fed an Oxalate-rich diet. The heatmap and dendrogram display the z-scores of normalized counts for these genes. (C) Density ridge plots show gene expression distribution of core-enriched genes in enriched gene sets, with gradient color indicating adjusted p-values using the Benjamini-Hochberg method. (D) Top 10 enriched pathways from KEGG pathway analysis.

**Supplementary References**

1. Kirita Y, Wu H, Uchimura K, Wilson PC, Humphreys BD. Cell profiling of mouse acute kidney injury reveals conserved cellular responses to injury. Proc Natl Acad Sci U S A. 2020;117(27):15874-83.

2. Wolf FA, Angerer P, Theis FJ. SCANPY: large-scale single-cell gene expression data analysis. Genome Biol. 2018;19(1):15.

3. Lun AT, McCarthy DJ, Marioni JC. A step-by-step workflow for low-level analysis of single-cell RNA-seq data with Bioconductor. F1000Res. 2016;5:2122.

4. Korsunsky I, Millard N, Fan J, Slowikowski K, Zhang F, Wei K, et al. Fast, sensitive and accurate integration of single-cell data with Harmony. Nat Methods. 2019;16(12):1289-96.

5. Satija R, Farrell JA, Gennert D, Schier AF, Regev A. Spatial reconstruction of single-cell gene expression data. Nat Biotechnol. 2015;33(5):495-502.

6. Wolf FA, Hamey FK, Plass M, Solana J, Dahlin JS, Gottgens B, et al. PAGA: graph abstraction reconciles clustering with trajectory inference through a topology preserving map of single cells. Genome Biol. 2019;20(1):59.

7. Kim D, Paggi JM, Park C, Bennett C, Salzberg SL. Graph-based genome alignment and genotyping with HISAT2 and HISAT-genotype. Nat Biotechnol. 2019;37(8):907-15.

8. Li H, Handsaker B, Wysoker A, Fennell T, Ruan J, Homer N, et al. The Sequence Alignment/Map format and SAMtools. Bioinformatics (Oxford, England). 2009;25(16):2078-9.

9. Liao Y, Smyth GK, Shi W. featureCounts: an efficient general purpose program for assigning sequence reads to genomic features. Bioinformatics (Oxford, England). 2014;30(7):923-30.

10. Gaidatzis D, Burger L, Florescu M, Stadler MB. Analysis of intronic and exonic reads in RNA-seq data characterizes transcriptional and post-transcriptional regulation. Nat Biotechnol. 2015;33(7):722-9.

11. Yu G, Wang LG, Han Y, He QY. clusterProfiler: an R package for comparing biological themes among gene clusters. OMICS. 2012;16(5):284-7.

12. Mulay SR, Eberhard JN, Pfann V, Marschner JA, Darisipudi MN, Daniel C, et al. Oxalate-induced chronic kidney disease with its uremic and cardiovascular complications in C57BL/6 mice. Am J Physiol Renal Physiol. 2016;310(8):F785-F95.

13. Lech M, Grobmayr R, Ryu M, Lorenz G, Hartter I, Mulay SR, et al. Macrophage phenotype controls long-term AKI outcomes--kidney regeneration versus atrophy. J Am Soc Nephrol. 2014;25(2):292-304.

14. Zhao ZB, Marschner JA, Iwakura T, Li C, Motrapu M, Kuang M, et al. Tubular Epithelial Cell HMGB1 Promotes AKI-CKD Transition by Sensitizing Cycling Tubular Cells to Oxidative Stress: A Rationale for Targeting HMGB1 during AKI Recovery. J Am Soc Nephrol. 2023;34(3):394-411.

15. Kintscher U, Bakris GL, Kolkhof P. Novel non-steroidal mineralocorticoid receptor antagonists in cardiorenal disease. Br J Pharmacol. 2022;179(13):3220-34.
