# Supplementary figures and images for: Gasdermin D deficiency aggravates nephrocalcinosis-related chronic kidney disease with rendering macrophages vulnerable to necroptosis
Source: Cell Death Dis. 2025 Apr 13;16(1):283. doi: 10.1038/s41419-025-07620-1 (PMC11993636; doi:10.1038/s41419-025-07620-1)

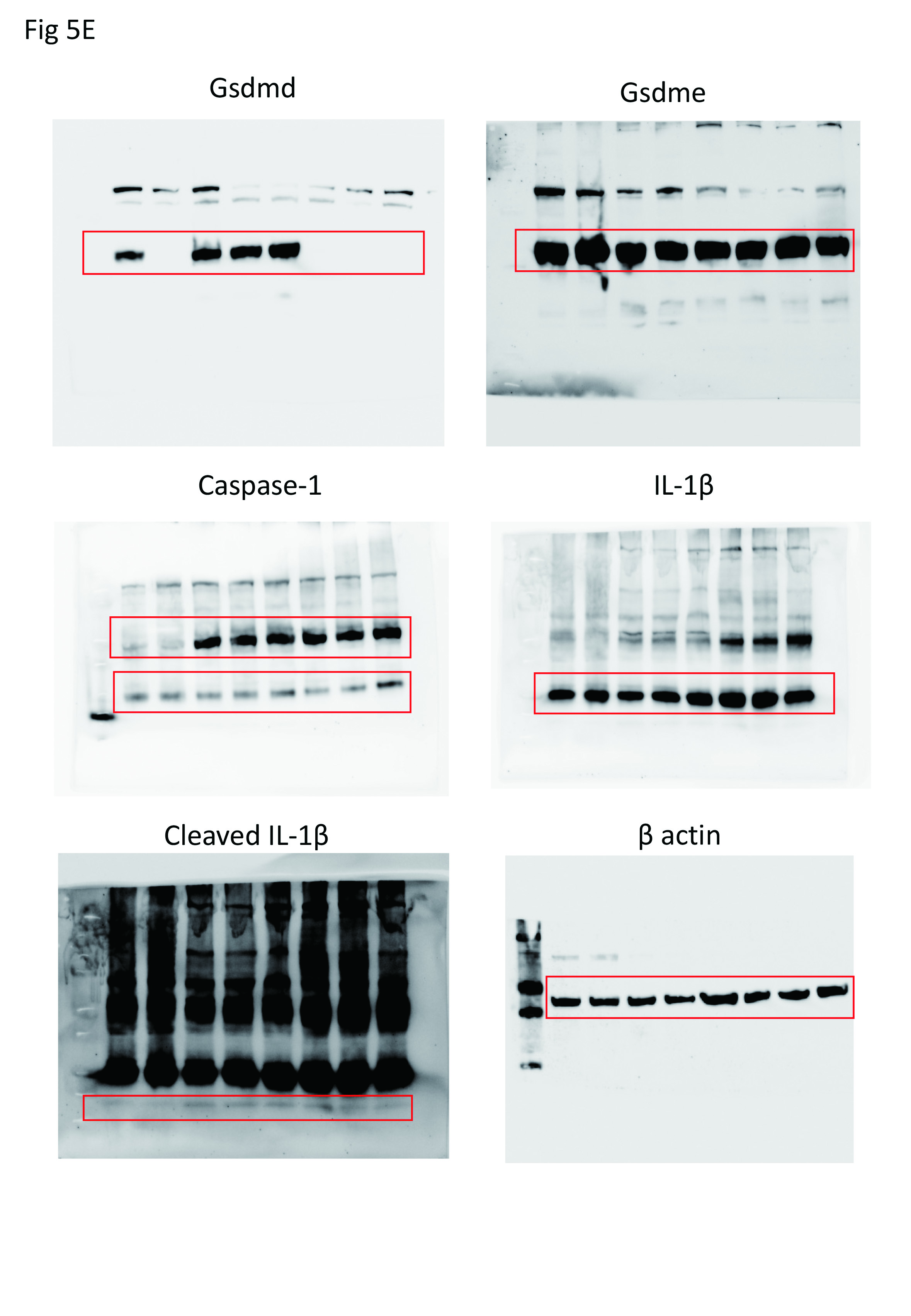

Supplement: Supplementary file 2 — original data [file 41419_2025_7620_MOESM2_ESM.jpg]

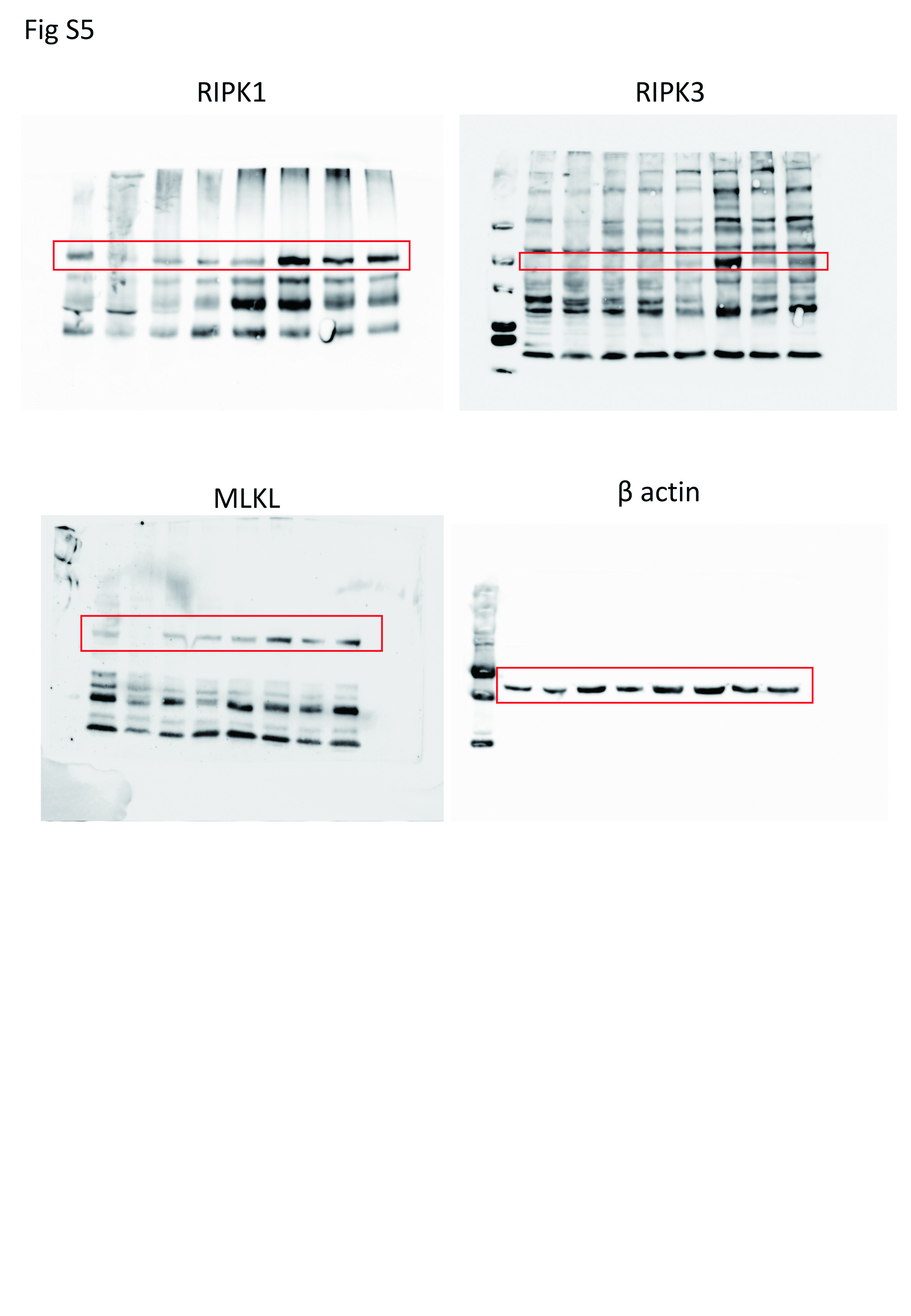

Supplement: Supplementary file 3 — original data [file 41419_2025_7620_MOESM3_ESM.jpg]
